# The AURKA inhibitor alters the immune microenvironment and enhances targeting B7-H3 immunotherapy in glioblastoma

**DOI:** 10.1172/jci.insight.173700

**Published:** 2025-02-10

**Authors:** Jinqiu Liu, Yuxuan Deng, Zhuonan Pu, Yazhou Miao, Zhaonian Hao, Herui Wang, Shaodong Zhang, Hanjie Liu, Jiejun Wang, Yifan Lv, Boyi Hu, Hong Wan, Zhengping Zhuang, Tai Sun, Shuyu Hao, Nan Ji, Jie Feng

**Affiliations:** 1 Beijing Neurosurgical Institute, Capital Medical University, Beijing, China.; 2 Department of Neurosurgery, Beijing Tiantan Hospital, Capital Medical University, Beijing, China.; 3 National Cancer Institute, NIH, Bethesda, Maryland, USA.

**Keywords:** Oncology, Therapeutics, Cancer immunotherapy

## Abstract

Glioblastoma (GBM) is one of the most lethal adult brain tumors with limited effective therapeutic options. Immunotherapy targeting B7-H3 (*CD276*) has shown promising efficacy in the treatment of gliomas. However, the response to this treatment varies among glioma patients due to individual differences. It’s necessary to find an effective strategy to improve the efficacy of targeting B7-H3 immunotherapy for nonresponders. In this study, we demonstrated a strong correlation between aurora kinase A (*AURKA*) and *CD276* expression in glioma tissue samples. Additionally, both *AURKA* knockdown and overexpression resulted in parallel changes in B7-H3 expression levels in glioma cells. Mechanistically, AURKA elevated B7-H3 expression by promoting epidermal growth factor receptor (EGFR) phosphorylation, which was validated in glioma cell lines and primary GBM cells. What’s more, the combination of AURKA inhibitor (alisertib) and anti–B7-H3 antibody markedly reduced tumor size and promoted CD8^+^ T cell infiltration and activation in mouse orthotopic syngeneic glioma models. To our knowledge, this study is the first to demonstrate AURKA-mediated B7-H3 upregulation in glioma cells; moreover, it proposes a promising therapeutic strategy combining the AURKA inhibitor alisertib with B7-H3–specific blocking mAbs.

## Abstract 2

<strong>Combination treatment with </strong><strong>an</strong><strong> AURKA inhibitor and anti-B7-H3 mAbs enhances antitumor efficacy through the EGFR/B7-H3 pathway and CD8^+^ T-cell infiltration in glioblastoma</strong>

## Introduction

Glioblastoma (GBM), categorized as a grade IV glioma by the World Health Organization (WHO), remains one of the most aggressive and lethal brain cancers in adults and is characterized by a low survival rate and a high rate of recurrence (1). Current standard treatments for GBM include surgical resection, chemotherapy, and radiotherapy. Despite these approaches, few effective therapeutic methods have been successfully developed to treat this cancer (1). As a result, identifying molecular targets and developing promising treatment strategies for GBM are critical.

Immunotherapies, such as immune checkpoint inhibitor therapy targeting programmed cell death receptor-1 ligand (PD-L1) and cytotoxic T lymphocyte–associated protein 4, have made remarkable progress in treating multiple cancers but have had minimal effect on GBM treatment (2, 3). Recently, the immune checkpoint molecule B7-H3 (*CD276*) has emerged as an immunotherapy target in GBM because of its high expression and limited heterogeneity in tumor cells, including GBM cells (3). B7-H3 promotes cancer progression and invasion through immunological and nonimmunological mechanisms (3). Various B7-H3–targeting immunotherapies have attracted attention with recent advances in molecular biology and antibody engineering (3, 4).

However, single-target B7-H3 immunotherapy in glioma has not been able to completely suppress tumor progression and has shown limited clinical efficacy (5). Therefore, effective strategies to enhance B7-H3–targeted immunotherapy are crucial. Previous studies have identified several cell cycle–related genes that regulate immune checkpoint expression, indicating that targeting the cell cycle with chemotherapy can influence immunotherapy efficacy (6–9). In this study, by analyzing the Chinese Glioma Genome Atlas (CGGA) and the Cancer Genome Atlas (TCGA) databases, we found that aurora kinase A (*AURKA*) expression is positively correlated with *CD276* expression. AURKA, a conserved serine/threonine kinase, is essential for regulating cell division during mitosis (10). *AURKA* is also regarded as an oncogene and is overexpressed in various types of cancers, contributing to tumor development (10, 11). Owing to its role in cancer, AURKA is considered a potential pharmacological target. Different specific AURKA inhibitors, including alisertib, have been reported and assessed in clinical trials (10, 11). Alisertib has been shown to induce aberrant cell cycle arrest in the G2/M phase, causing apoptosis and reprogramming of tumor immune microenvironments in various types of cancer cells (11, 12). Alisertib has demonstrated efficacy in treating different solid tumors in vitro (11). Clinical trials have further revealed that alisertib has therapeutic effects on various types of solid tumors (10, 11). Importantly, alisertib can pass through the blood-brain barrier, making it a promising therapeutic option for central nervous system–associated malignancies (11, 13–15).

Previous studies have shown that Aurora kinases can regulate the epidermal growth factor receptor (EGFR) signaling pathway. AURKA binds to syndecan binding protein (SDCBP) and phosphorylates it, inhibiting the ubiquitination-mediated degradation of SDCBP. The accumulated SDCBP activates EGFR by binding to it and preventing its internalization (16). Under normal conditions, EGFR is located on the cell surface in a structurally compact autoinhibited state with minimal kinase activity (17, 18). After EGFR is activated, it can promote the expression of tumor immune checkpoints, such as PD-L1, tumor cell proliferation, angiogenesis, and invasion, through its downstream effectors, including the phosphoinositide 3-kinase/protein kinase B, mitogen-activated protein kinase, rat sarcoma virus/rapidly accelerated fibrosarcoma/mitogen-activated extracellular signal-regulated kinase/extracellular signal-regulated kinase, Janus kinase/signal transducer and activator of transcription, and phospholipase C γ 1/protein kinase C pathways (17, 19).

In this study, we investigated whether AURKA regulates the expression of B7-H3 in GBM cells through EGFR phosphorylation and, as a result, increases the responsiveness of GBM cells to B7-H3–specific blocking mAbs. Moreover, we compared the ability of combination treatment with alisertib and B7-H3–specific blocking mAbs with that of treatments alone to improve the immune microenvironment of GBM and inhibit tumor progression. Our research aims to elucidate the underlying mechanism of AURKA-mediated B7-H3 upregulation and explore the potential of combining the AURKA inhibitor alisertib with B7-H3–specific blocking mAbs as a promising therapeutic strategy for GBM treatment.

## Results

### Increased B7-H3 expression in GBM and the correlation between AURKA and B7-H3.

**Figure 1:**
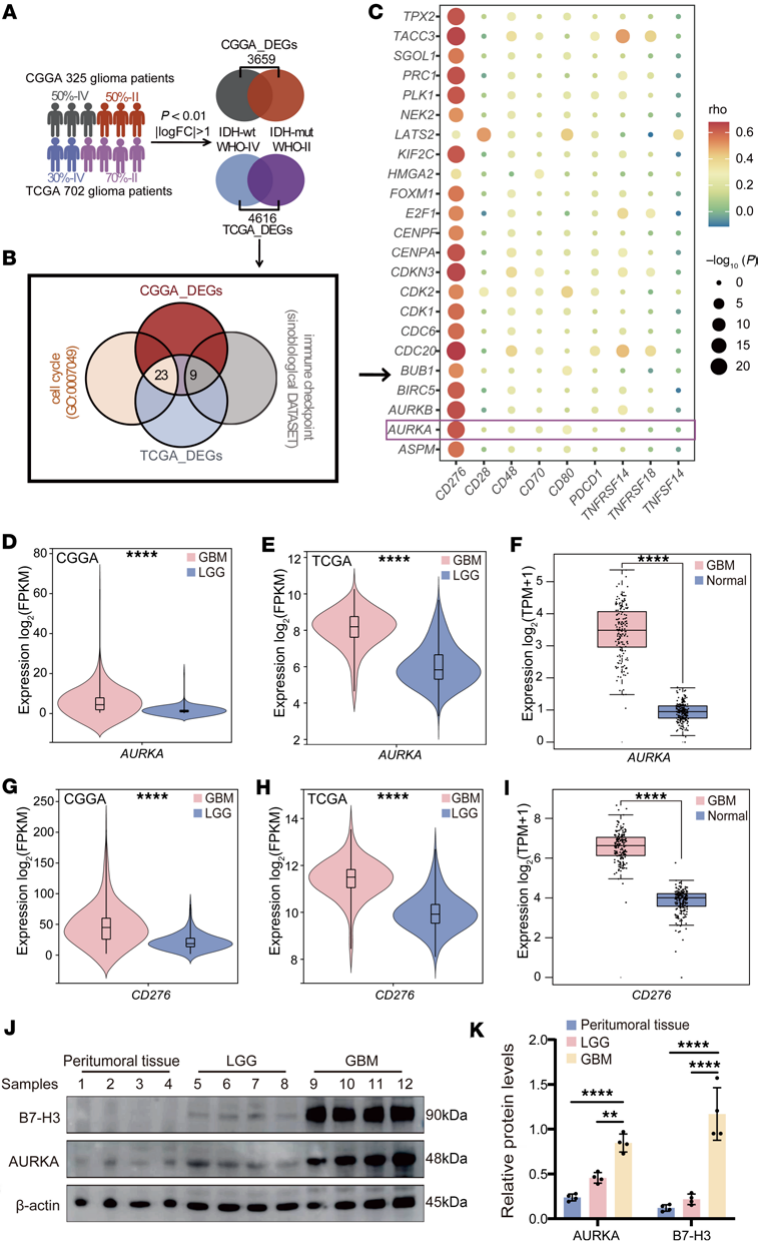
Correlation of AURKA with *CD276* in GBM. (**A**) The filtering process of the DEGs between GBM and LGG in the TCGA and CGGA datasets. (**B**) Venn diagrams displaying the overlap of gene sets within the cell cycle and immune checkpoint pathways, respectively, on the basis of the DEGs in both the TCGA and CGGA cohorts. (**C**) Heatmaps displaying the correlations in the CGGA datasets between cell cycle genes among the DEGs in both the TCGA and CGGA cohorts and immune checkpoint genes among the DEGs in both the TCGA and CGGA cohorts. (**D** and **E**) *AURKA* mRNA expression in GBM (*n* = 190 for CGGA, *n* = 142 for TCGA) and LGG (*n* = 135 for CGGA, *n* = 419 for TCGA) samples from the CGGA dataset (**D**) and TCGA dataset (**E**). (**F**) *AURKA* mRNA expression in GBM (*n* = 163) and normal brain tissues (*n* = 207) from TCGA datasets and the GTEx database. (**G** and **H**) *CD276* mRNA expression in GBM (*n* = 190 for CGGA, *n* = 142 for TCGA) and LGG (*n* = 135 for CGGA, *n* = 419 for TCGA) samples from the CGGA dataset (**G**) and TCGA dataset (**H**). (**I**) *CD276* mRNA expression in GBM (*n* = 163) and normal brain tissues (*n* = 207) from TCGA datasets and the GTEx database. (**J** and **K**) Western blot analysis of AURKA and B7-H3 expression in peritumoral, LGG, and GBM samples (*n* = 4/group) with quantification (**K**); β-actin was used as the internal control. Statistical significance was assessed via 2-tailed unpaired Student’s *t* test (**D**, **E**, **G**, and **H**) and 1-way ANOVA followed by Tukey’s multiple-comparison test (**K**). The data are presented as the mean ± SD (**D**, **E**, **G**, **H**, and **K**). **P < 0.01, ****P < 0.0001.

First, we investigated the differentially expressed genes (DEGs) between GBM (WHO-IV and isocitrate dehydrogenase [*IDH*] WT) and low-grade gliomas (LGG, WHO-II, and IDH mutant) via the TCGA and CGGA databases (Figure 1A). We identified 4,616 DEGs in the TCGA database and 3,659 DEGs in the CGGA database (*P* < 0.01 and |log [fold change]| > 1) (Figure 1A and Supplemental Figure 1, A and B; supplemental material available online with this article; https://doi.org/10.1172/jci.insight.173700DS1). We performed an intersection analysis between the DEGs identified in both the TCGA and CGGA databases and the genes annotated under the Gene Ontology (GO) term for cell cycle (accession no. GO:0007049), resulting in the identification of 23 DEGs that were part of the cell cycle gene set (Figure 1B). Furthermore, these DEGs identified in both the TCGA and CGGA databases were intersected with immune checkpoint genes from the Sinobiological Database (https://www.sinobiological.com/category/immunecheckpoint-proteins-list). We identified 9 genes that were in the immune checkpoint gene set (Figure 1B). Moreover, we assessed the correlation between the 23 DEGs in the cell cycle gene set and the 9 DEGs in the immune checkpoint gene set in GBM (Figure 1C and Supplemental Figure 1C). Our data reveal that the inhibitory immune checkpoint *CD276* was significantly correlated with most of the DEGs identified in the cell cycle gene set, including *AURKA*. Additionally, our findings revealed a fold change of approximately 3.03 for *AURKA* between GBM and LGG in the CGGA database (Figure 1D; *P* < 0.0001). In the TCGA database, the fold change in *AURKA* expression between patients with GBM and those with LGG was approximately 5.00 (Figure 1E; *P* < 0.0001). Furthermore, we conducted a comparative analysis using both the TCGA GBM database and Genotype-Tissue Expression Project (GTEx) data to examine differences in *AURKA* expression between GBM and normal brain tissues. Our results reveal a significant difference in *AURKA* expression between GBM and normal brain tissues (*P* < 0.0001, fold change of approximately 5.80; Figure 1F). Similarly, *CD276* was more highly expressed in GBM than in LGG (Figure 1, G and H). Additionally, *CD276* expression was elevated in tumor tissue compared with normal brain tissue (Figure 1I).

To further validate this observation, we performed Western blotting analysis on 4 LGG tissue samples and GBM tissue samples in comparison with 4 peritumoral samples. We confirmed increased B7-H3 and AURKA protein expression in tissue samples obtained from 4 patients with GBM compared with those obtained from 4 patients with LGG and 4 peritumoral samples via Western blotting (Figure 1, J and K).

### AURKA regulates B7-H3 expression through EGFR activation.

**Figure 2:**
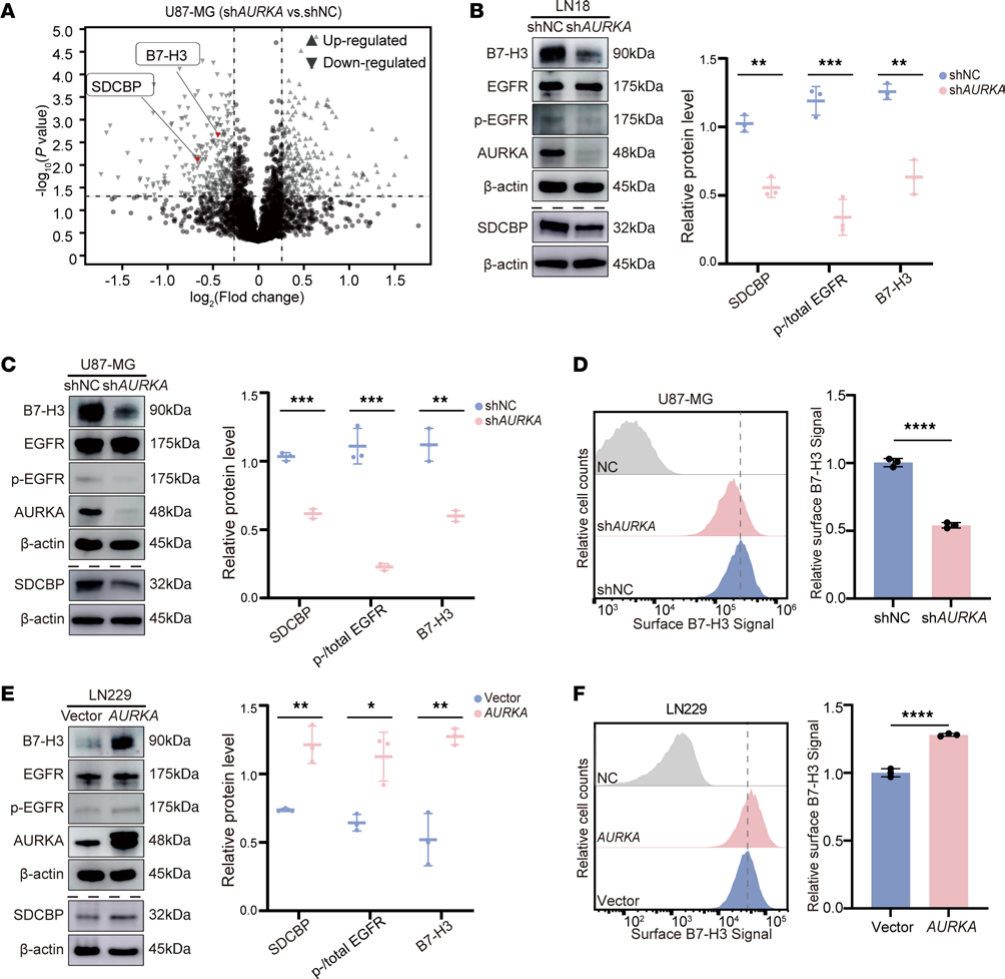
AURKA regulates B7-H3 expression. (**A**) Volcano plot of the proteomics data showing reduced expression of B7-H3 and SDCBP (red dots) in U87-MG cells treated with shRNA targeting *AURKA*. (**B** and **C**) The protein expression levels of B7-H3, total AURKA, SDCBP, p-EGFR (Y1068), and total EGFR in LN18 cells (**B**) and U87-MG cells (**C**) expressing shNC or *AURKA*-shRNA#3 were detected via Western blotting, and the quantifications are shown on the right. β-Actin was used as the internal control. (**D**) B7-H3 expression on the cell surface in U87-MG cells expressing shNC or *AURKA*-shRNA#3 was detected by flow cytometry, and the quantification of the results is shown on the right. Cells that were only stained with isotype control antibodies were used as the negative control (NC). (**E**) The protein expression levels of B7-H3, total AURKA, SDCBP, p-EGFR (Y1068), and total EGFR in LN229 cells expressing vector or *AURKA*_cDNA_Flag were detected via Western blotting, and the quantifications are shown on the right. β-Actin was used as the internal control. (**F**) B7-H3 expression on the cell surface in LN229 cells expressing vector or *AURKA*_cDNA_Flag was detected by flow cytometry, and the quantification of the results is shown on the right. Cells that were only stained with isotype control antibodies were used as the negative control (NC). All samples were biologically independent, and 3 independent experiments were performed. Comparisons were performed via 1-tailed unpaired Student’s *t* test (**A**) or 2-tailed unpaired Student’s *t* test (**B**–**F**). The data are presented as the mean ± SD (**B**–**F**). *P < 0.05, **P < 0.01, ***P < 0.001, ****P < 0.0001.

Since our results demonstrate that *AURKA* expression was positively correlated with *CD276* expression, we explored whether AURKA regulates B7-H3 expression. First, AURKA expression was detected in the human glioma cell lines LN18, U87-MG, U373, LN229, TJ906, M059K, and U251 (Supplemental Figure 2A), which were used as in vitro models. LN229 cells, which express low levels of AURKA, were used to overexpress AURKA with *AURKA*_cDNA_Flag (Supplemental Figure 2B). LN18 and U87-MG cells that expressed high levels of AURKA were used to silence *AURKA* via specific shRNA#1, shRNA#2 or shRNA#3, with shRNA#3 significantly decreasing AURKA expression in both LN18 and U87-MG cells (Supplemental Figure 2, C and D). Furthermore, we analyzed the proteomics profiling of GBM cell lines U87_MG treated with or without shRNA#3 targeting *AURKA*. Proteomic analysis revealed that B7-H3 expression was lower in the shAURKA#3 groups than in the shNC groups in the U87-MG cell line (Figure 2A). And we confirmed that silencing of AURKA (sh*AURKA*#3) led to a significant decrease in the B7-H3 protein level in LN18 and U87-MG cells (Figure 2, B and C) and in the *CD276* mRNA expression level in LN18 cells (Supplemental Figure 2E). Additionally, B7-H3 expression on the cell membrane decreased after AURKA was knocked down in U87-MG cells (Figure 2D). Similarly, knockdown of AURKA (si*AURKA*) significantly decreased B7-H3 protein expression in LHG cell lines (Supplemental Figure 2F). In contrast, overexpression of AURKA significantly increased *CD276* protein and mRNA expression levels in LN229 cells (Figure 2, E and F, and Supplemental Figure 2G). Several studies have demonstrated the association between the upregulation of B7-H3 and EGFR signaling (20, 21). Thus, we speculated that AURKA regulates B7-H3 expression through EGFR signaling. The phosphorylation of EGFR at Try1068 (pEGFR/EGFR) changed significantly after the knockdown or overexpression of AURKA in U87-MG, LN18, and LN229 cells, whereas the total EGFR expression level did not change significantly (Figure 2, B, C, and E).

Previously, AURKA was shown to promote EGFR phosphorylation by inhibiting the degradation of SDCBP (16). Our proteomics analysis also demonstrated that inhibition of AURKA by shRNA decreases SDCBP protein levels (Figure 2A). We confirmed that silencing AURKA (sh*AURKA*#3) resulted in a significant reduction in the SDCBP protein in LN18 and U87-MG cells (Figure 2, B and C). Consistently, overexpression of AURKA significantly increased the level of the SDCBP protein in LN229 cells (Figure 2E).

**Figure 3:**
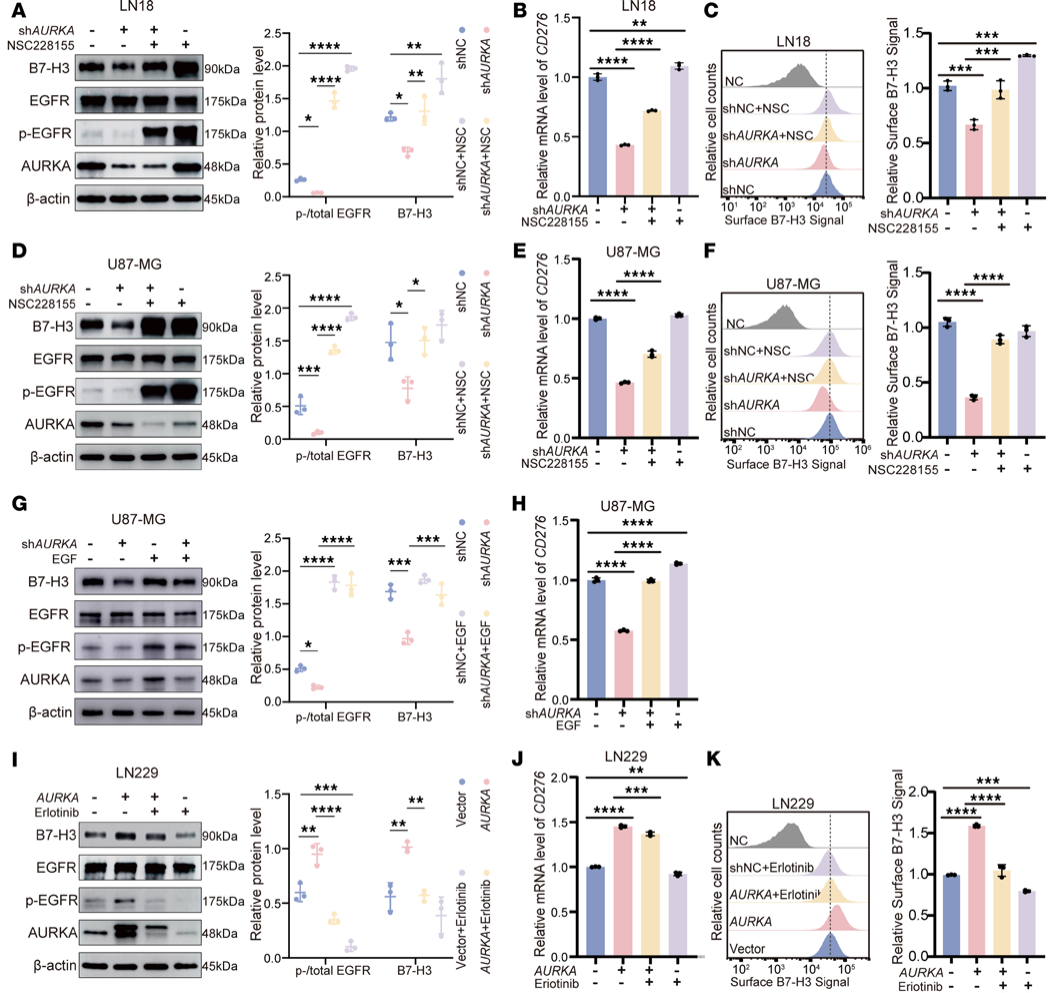
AURKA regulates B7-H3 expression through EGFR phosphorylation. (**A**–**F**) The expression levels of various markers were analyzed in LN18 cells (**A**–**C**) and U87-MG cells (**D**–**F**) expressing shNC or *AURKA*-shRNA#3 treated with NSC228155 (10 μM, 8 hours) or DMSO. (**A** and **D**) Protein expression of B7-H3, total AURKA, p-EGFR (Y1068), and total EGFR was assessed by Western blotting. (**B** and **E**) The mRNA level of *CD276* was measured by qPCR. (**C** and **F**) B7-H3 expression on the cell surface was analyzed using flow cytometry. (**G** and **H**) The expression levels of various markers were analyzed in U87-MG cells expressing shNC or *AURKA*-shRNA#3 with or without EGF (24 hours, 500 ng/mL). (**G**) Protein expression of B7-H3, total AURKA, p-EGFR (Y1068), and total EGFR was assessed by Western blotting. (**H**) The mRNA level of *CD276* was measured by qPCR. (**I**–**K**) The expression levels of various markers were analyzed in LN229 cells expressing vector or *AURKA*_cDNA_Flag with erlotinib (24 hours, 60 μM) or DMSO. (**I**) Protein expression of B7-H3, total AURKA, p-EGFR (Y1068), and total EGFR was assessed by Western blotting. (**J**) The mRNA level of *CD276* was measured by qPCR. (**K**) B7-H3 expression on the cell surface was analyzed using flow cytometry. The quantifications are shown on the right (**A**, **C**, **D**, **F**, **G**, **I**, and **K**). β-Actin was used as the internal control (**A**, **D**, **G**, and **I**). Cells that were only stained with isotype control antibodies were used as the negative control (NC) (**C**, **F**, and **K**). Statistical significance was assessed by 1-way ANOVA followed by Tukey’s multiple-comparison test (**A**–**K**). The data are presented as the mean ± SD (**A**–**K**). All samples were biologically independent, and 3 independent experiments were performed. *P < 0.05, **P < 0.01, ***P < 0.001, ****P < 0.0001.

To determine whether the phosphorylation of EGFR is the true effector of AURKA-mediated B7-H3 expression, the EGFR activator NSC228155 or epidermal growth factor (EGF) was used in AURKA-knockdown cells. Treatment with 10 μM NSC228155 for 8 hours significantly increased the phosphorylation of EGFR at Tyr1068 (pEGFR/EGFR) (Figure 3, A and D), which was followed by an increase in *CD276* protein and mRNA levels after AURKA knockdown in the LN18 and U87-MG cell lines (Figure 3, A–F). Similarly, treatment with 500 ng/mL EGF for 24 hours also increased the phosphorylation of EGFR at Tyr1068 (pEGFR/EGFR) and increased *CD276* mRNA and protein expression levels after AURKA knockdown in U87-MG cell lines (Figure 3, G and H).

In contrast, treatment with the EGFR inhibitor erlotinib (60 μM) for 24 hours suppressed the phosphorylation of EGFR at Tyr1068 (pEGFR/EGFR) and was accompanied by decreases in *CD276* protein and mRNA expression levels in AURKA-overexpressing LN229 cells (Figure 3, I–K).

These findings confirm that EGFR phosphorylation plays a functional role in AURKA-mediated B7-H3 expression. The effect of AURKA on the activation of EGFR may result from maintaining the stability of SDCBP.

### Alisertib inhibits glioma cell proliferation and induces B7-H3 expression via EGFR activation.

Previous studies have demonstrated that alisertib, a clinically validated specific AURKA inhibitor, suppresses the growth of various glioma cell lines (22). To assess the cytotoxic effects of different concentrations of alisertib on glioma cells, we measured the viability of U87-MG and LN18 cells after 72 hours of alisertib treatment. Our findings revealed that alisertib treatment substantially inhibited the proliferation of U87-MG and LN18 cells in a dose-dependent manner. The 50% inhibitory concentration (IC_50_) of alisertib for U87-MG cells was 7.71 μM; the IC_50_ for LN18 cells was 1.64 μM (Supplemental Figure 3A).

**Figure 4:**
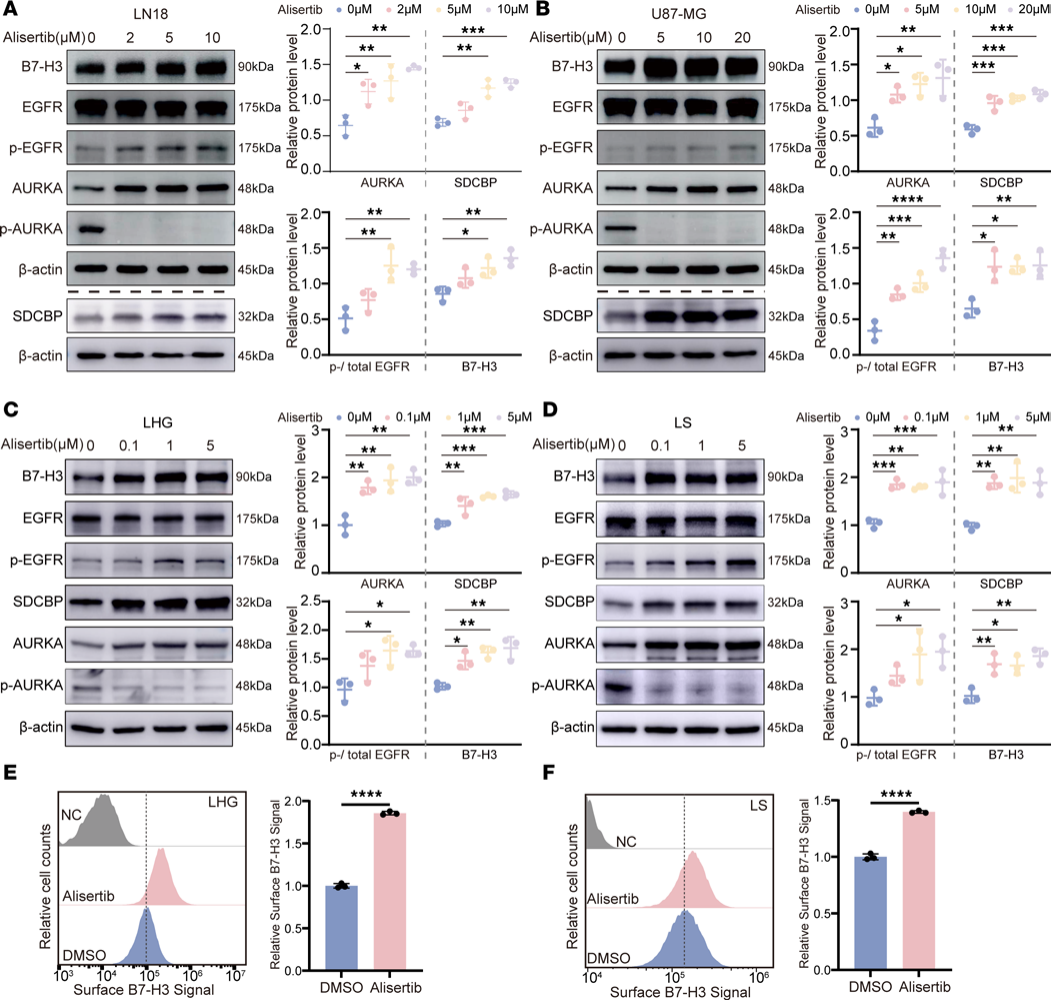
The AURKA inhibitor upregulates B7-H3 expression. (**A**–**D**) The protein expression levels of B7-H3, SDCBP, total AURKA, p-AURKA (T288), and p-EGFR (Y1068) and the total EGFR in LN18 (**A**), U87-MG (**B**), LHG (**C**) and LS (**D**) cells treated with increasing concentrations of alisertib for 24 hours were detected via Western blotting, and the quantifications are shown on the right. β-Actin was used as the internal control. (**E** and **F**) B7-H3 on the cell surface of LHG (**E**) and LS (**F**) cells treated with alisertib (24 hours, 0.1 μM) or DMSO was detected via flow cytometry, and the quantification of the results is shown on the right. Cells that were only stained with isotype control antibodies were used as the negative control (NC). Statistical significance was assessed by 1-way ANOVA followed by Tukey’s multiple-comparison test (**A**–**D**) and by a 2-tailed unpaired Student’s *t* test (**E** and **F**). The data are presented as the mean ± SD (**A**–**F**). All samples were biologically independent, and 3 independent experiments were performed. *P < 0.05, **P < 0.01, ***P < 0.001, ****P < 0.0001.

Our results revealed that alisertib treatment upregulated B7-H3 protein expression by activating EGFR signaling in glioma cells. First, we confirmed that alisertib treatment increased B7-H3, SDCBP, and phosphorylated EGFR expression in glioma cells (Figure 4, A–F, and Supplemental Figure 3, B–D). Treatment with alisertib eliminated autophosphorylation of AURKA at Thr288 (T288), accompanied by a dramatic increase in *AURKA* protein and mRNA levels (Figure 4, A–D, and Supplemental Figure 3, C–F). The same results were observed in 293T cells, where upregulated B7-H3 expression was observed after treatment with alisertib (Supplemental Figure 3, G and H). However, there was no significant change in B7-H3 expression in normal human astrocyte (NHA) cells (Supplemental Figure 3, I and J), suggesting that alisertib treatment upregulated B7-H3 expression in tumor cells but not NHA cells.

**Figure 5:**
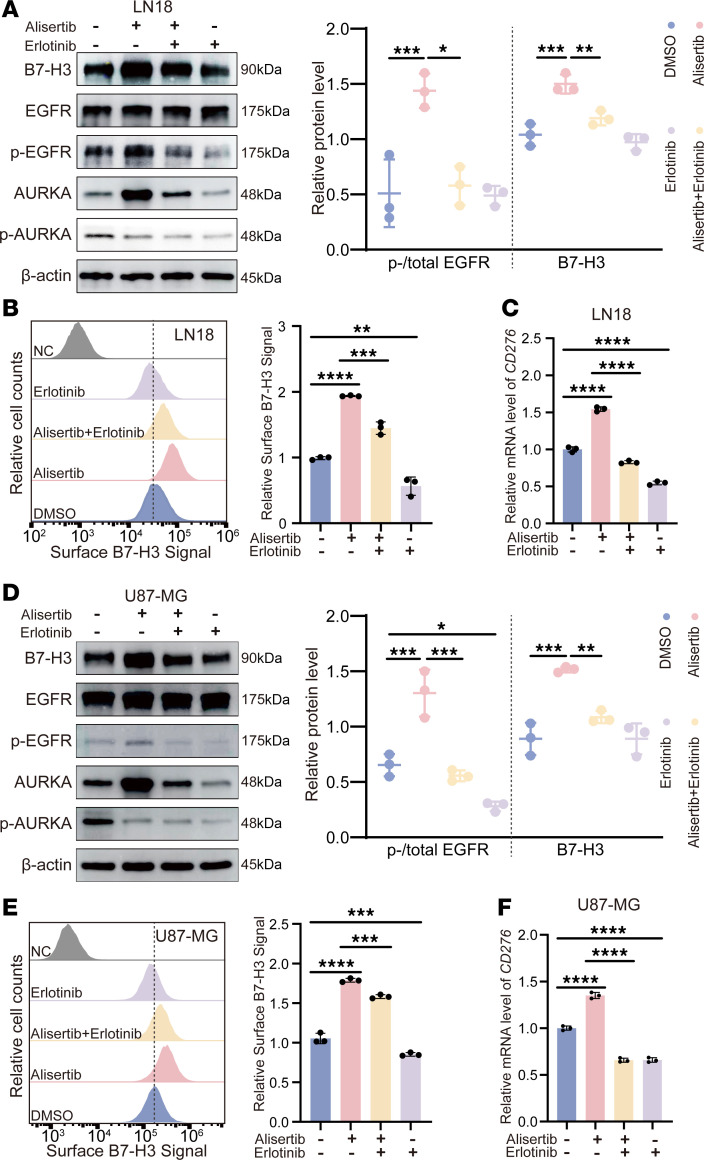
The AURKA inhibitor regulates B7-H3 expression through EGFR phosphorylation. (**A**) The protein expression of B7-H3, p-AURKA (T288), total AURKA, p-EGFR (Y1068), and total EGFR in LN18 cells treated with alisertib (24 hours, 5 μM), erlotinib (24 hours, 60 μM), or both was detected by Western blotting. (**B**) B7-H3 expression on the cell surface of LN18 cells treated with alisertib (24 hours, 5 μM), erlotinib (24 hours, 60 μM), or both was detected via flow cytometry, and the quantifications are shown on the right. Cells that were only stained with isotype control antibodies were used as the negative control (NC). (**C**) The mRNA level of *CD276* in LN18 cells treated with alisertib (24 hours, 5 μM), erlotinib (24 hours, 60 μM), or both. (**D**) The protein expression levels of B7-H3, p-AURKA (T288), total AURKA, p-EGFR (Y1068), and total EGFR in U87-MG cells treated with alisertib (24 hours, 5 μM), erlotinib (24 hours, 60 μM), or both were detected by Western blotting. (**E**) B7-H3 expression on the surface of U87-MG cells treated with alisertib (24 hours, 5 μM), erlotinib (24 hours, 60 μM), or both was detected by flow cytometry, and quantifications are shown on the right. Cells that were only stained with isotype control antibodies were used as the negative control (NC). (**F**) The mRNA level of *CD276* in U87-MG cells treated with alisertib (24 hours, 5 μM), erlotinib (24 hours, 60 μM), or their combination. The quantifications are shown on the right (**A** and **D**). β-Actin was used as the internal control (**A** and **D**). Statistical significance was assessed by 1-way ANOVA followed by Tukey’s multiple-comparison test (**A**–**F**). The data are presented as the mean ± SD (**A**–**F**). All samples were biologically independent, and 3 independent experiments were performed. *P < 0.05, **P < 0.01, ***P < 0.001, ****P < 0.0001.

Next, we investigated how alisertib treatment increases B7-H3 protein levels. On the basis of these previous findings, we hypothesized that alisertib affects EGFR activation, which regulates B7-H3 protein levels. To test this hypothesis, we disrupted EGFR activation by administering the EGFR inhibitor erlotinib. We treated LN18 and U87-MG cells with alisertib in the presence or absence of erlotinib. Our results showed that alisertib increased AURKA expression and EGFR phosphorylation at Tyr1068 without erlotinib (Figure 5, A and D). Following alisertib treatment, the cells were treated with 60 μM erlotinib for 24 hours. As expected, erlotinib treatment reduced the phosphorylation of EGFR at Tyr1068 and decreased *CD276* protein and mRNA levels in LN18 and U87-MG cells (Figure 5, A–F).

In conclusion, we demonstrated that alisertib treatment elevated B7-H3 expression in glioma cells by increasing EGFR activity. Therefore, we propose that the on-target effect of alisertib on the activation of EGFR may be attributed to its ability to increase AURKA expression, thereby maintaining SDCBP stability and subsequently activating the EGFR pathway.

### AURKA inhibitors modulate B7-H3 and the immune microenvironment.

**Figure 6:**
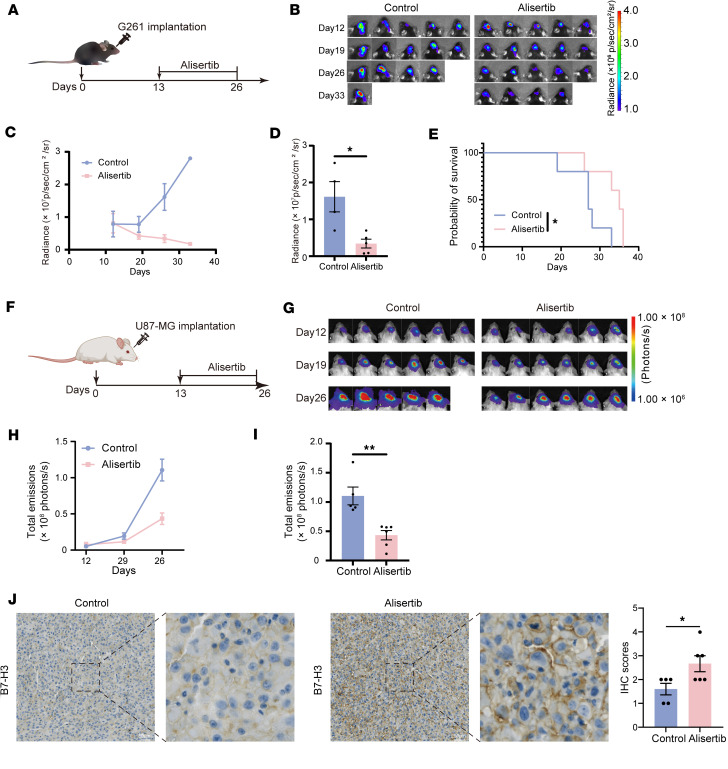
AURKA inhibitors suppress tumor growth and upregulate B7-H3 expression. (**A**) Schematic diagram of the in vivo medication studies in G261-bearing mice. Alisertib or vehicle was administered daily on day 13 after tumor inoculation for 2 weeks. (**B** and **C**) Changes in the volume of orthotopic G261 tumors treated with or without alisertib at various time points were measured via the IVIS system (*n* = 5/group) and quantified. (**D**) The volume of orthotopic G261 tumors treated with or without alisertib on day 26 was measured via the IVIS system. (*n* = 5 for the alisertib group, *n* = 4 for the control group). (**E**) Kaplan-Meier survival plots of mice bearing orthotopic G261 tumors treated with or without alisertib (*n* = 5/group). (**F**) Schematic diagram of the in vivo medication studies in U87-MG–bearing mice. Alisertib or vehicle was administered daily on day 13 after tumor inoculation for 2 weeks. (**G** and **H**) Changes in the volume of orthotopic U87-MG tumors treated with or without alisertib at various time points were measured via the IVIS system (*n* = 6/group) and quantified. (**I**) The volume of orthotopic U87-MG tumors treated with or without alisertib on day 26 was measured via the IVIS system (*n* = 6 for the alisertib group, *n* = 5 for the control group). (**J**) IHC analysis of B7-H3 in orthotopic U87-MG tumors treated with or without alisertib; the quantifications are shown on the right (*n* = 6 for the alisertib group, *n* = 5 for the control group). Statistical significance was assessed by using a 2-tailed unpaired Student’s *t* test (**D**, **I**, and **J**) and the log-rank (Mantel-Cox) test (**E**). The data are presented as the mean ± SEM (**C**, **D**, **H**, **I**, and **J**). *P < 0.05, **P < 0.01. Scale bars: 50 µm.

We then assessed whether our observations had translational relevance. First, we investigated whether alisertib inhibited proliferation and increased B7-H3 expression in G261 mouse glioma cells at different concentrations and time points. The results showed that cell viability was substantially suppressed by alisertib treatment in a dose-dependent manner at 72 hours (Supplemental Figure 4A). B7-H3 expression in G261 cells was upregulated in a time- and dose-dependent manner (Supplemental Figure 4, B and C). We subsequently used a mouse syngeneic glioma model with G261-Luc cells to assess the efficacy of AURKA inhibition in vivo. Tumor-bearing mice were randomized into (a) the control and (b) alisertib groups. The tumor volume of C57BL/6 mice was detected via an in vivo imaging system (IVIS) at different time points (days 12, 19, 26, and 33 after inoculation with tumor cells). Alisertib was administered orally daily for 2 weeks. The time points of administration are shown in Figure 6A. We detected reduced tumor growth in the alisertib group compared with the control group after 2 weeks of alisertib treatment (Figure 6, B–D). Kaplan-Meier analysis of animal survival revealed that, compared with the control group, the alisertib group presented significantly longer survival times (*P* = 0.0487; Figure 6E).

Subsequently, we assessed the effect of AURKA inhibitors on the expression of B7-H3 in vivo through an orthotopic xenograft model employing U87-MG–Luc cells. Tumor-bearing mice were randomized into (a) the control and (b) alisertib groups. The tumor volume of NSG mice was detected via IVIS at different time points (12, 19, and 26 days after inoculation with tumor cells). Alisertib was administered orally daily to the mice in the alisertib group for 2 weeks, and the time points of administration are shown in Figure 6F (the schematics are drawn in the MedPeer platform; https://image.medpeer.cn/). We detected reduced tumor growth in the alisertib group compared with the control group after 2 weeks of alisertib treatment (Figure 6, G–I). We used IHC to analyze B7-H3 expression in control and alisertib-treated tumors. Tumors in the alisertib group presented significantly higher levels of B7-H3 expression than did those in the control group (Figure 6J).

**Figure 7:**
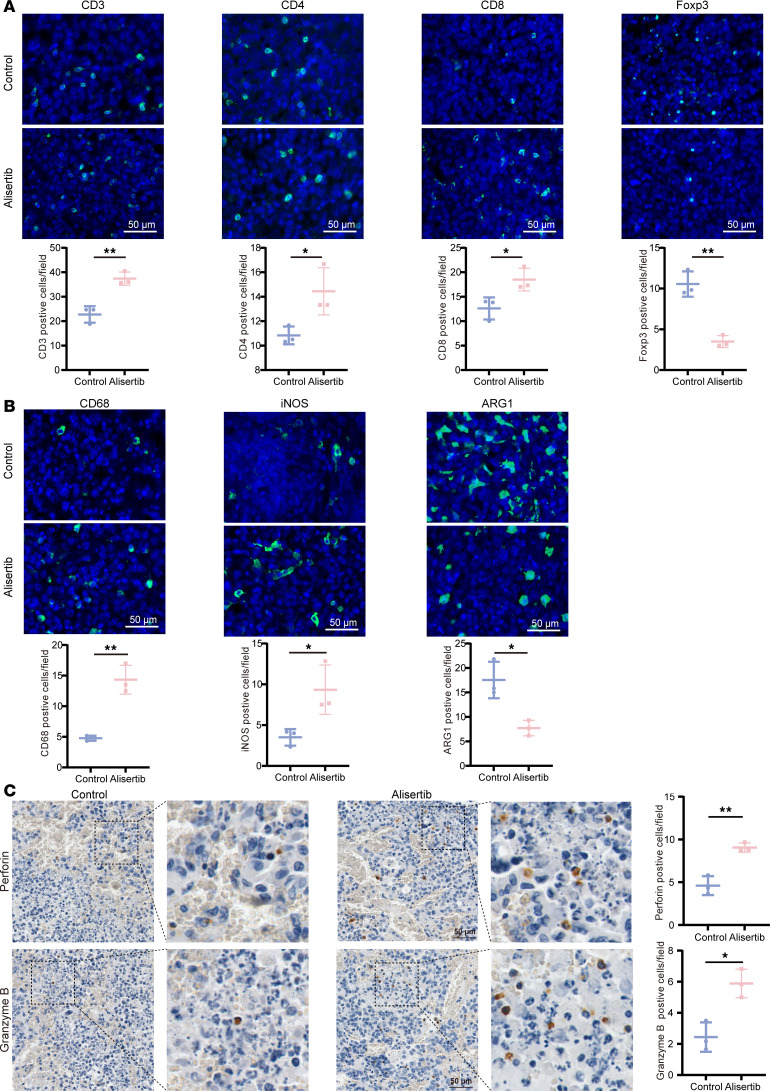
AURKA inhibitors affect immune cell infiltration and activation in glioma. (**A** and **B**) A panel of immune markers in orthotopic G261 tumors treated with or without alisertib were detected via immunofluorescence analysis, and the quantifications are presented below (*n* = 3/group). The T cell markers CD3, CD4, CD8, and Foxp3 (**A**). The macrophage markers CD68, iNOS, and ARG1 (**B**). (**C**) Perforin and granzyme B in orthotopic G261 tumors treated with or without alisertib were detected by IHC analysis, and the quantifications are shown on the right (*n* = 3/group). Statistical significance was assessed by using a 2-tailed unpaired Student’s *t* test (**A**–**C**). The data are presented as the mean ± SD (**A**–**C**). *P < 0.05, **P < 0.01. Scale bars: 50 µm.

To analyze tumor-infiltrating T lymphocytes and myeloid cells, we performed immunofluorescence and IHC staining on control and alisertib-treated tumors. Notably, the infiltration of CD3^+^, CD4^+^, and CD8^+^ T cells was considerably greater in alisertib-treated tumors than in control tumors (Figure 7A). In contrast, the number of Foxp3^+^ T cells, which are Tregs, was significantly lower in the alisertib-treated tumors than in the control tumors (Figure 7A). Furthermore, we observed increased numbers of CD68^+^ and iNOS^+^ cells, indicative of M1 (inflammatory) phenotype macrophages, after 2 weeks of alisertib treatment (Figure 7B). Conversely, the number of ARG1^+^ cells, which are M2 antiinflammatory macrophages, was notably lower in the alisertib-treated tumors than in the control tumors (Figure 7B). In addition, tumors in the alisertib group also had significantly more perforin^+^ and granzyme B^+^ cells than did those in the control group (Figure 7C). These findings suggest that AURKA inhibition enhances the antitumor immune response in glioma orthotopic syngeneic models.

### Combining alisertib with an anti–B7-H3 mAb reduces tumor size and increases CD8^+^ T cell infiltration.

**Figure 8:**
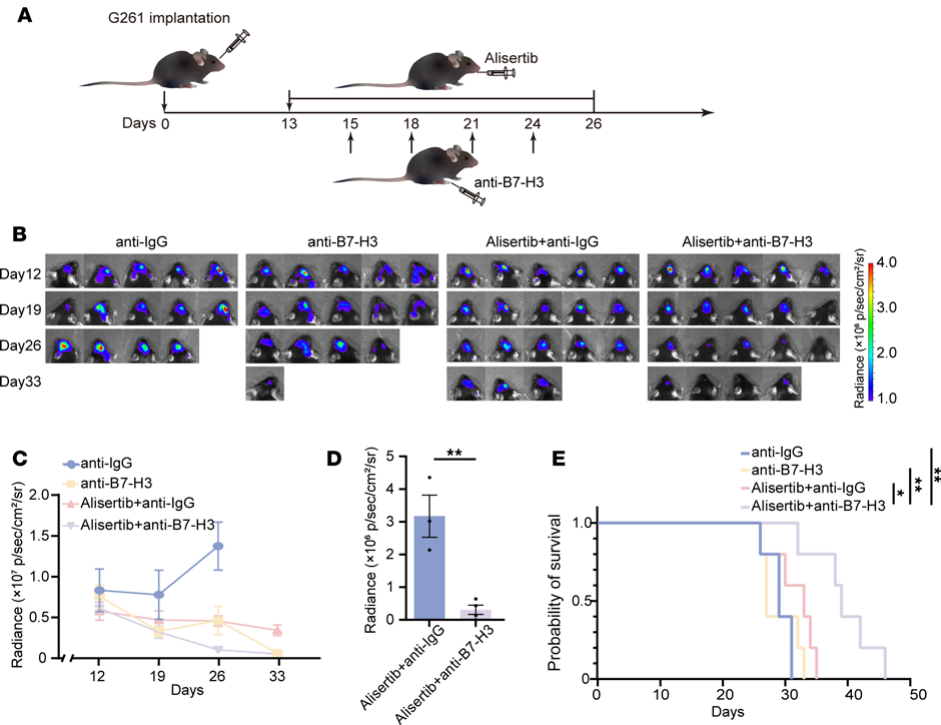
The combination of alisertib with an anti–B7-H3 mAb reduces tumor size. (**A**) Schematic diagram of the in vivo combination treatment in G261-bearing mice. The mice were treated with alisertib (daily × 14 days), B7-H3 mAbs (300 μg/injection × 4) or an isotype control antibody (300 μg/injection × 4) in combination. (**B** and **C**) Changes in the volume of orthotopic G261 tumors in the anti-IgG, alisertib + anti-IgG, anti–B7-H3, and alisertib + anti–B7-H3 groups were measured via the IVIS system (*n* = 5/group) and quantified. (**D**) The volume of orthotopic G261 tumors in the alisertib + anti-IgG and alisertib + anti–B7-H3 groups on day 33 was measured via the IVIS system (*n* = 3 for the alisertib + anti-IgG group, *n* = 4 for the alisertib + anti–B7-H3 group). (**E**) Kaplan-Meier survival plots of mice bearing orthotopic G261 tumors in the anti-IgG, alisertib + anti-IgG, anti–B7-H3, and alisertib + anti–B7-H3 groups (*n* = 5/group). Statistical significance was assessed via 2-tailed unpaired Student’s *t* test (**D**) and the log-rank (Mantel-Cox) test (**E**). The data are presented as the mean ± SEM (**C** and **D**). *P < 0.05; **P < 0.01.

**Figure 9:**
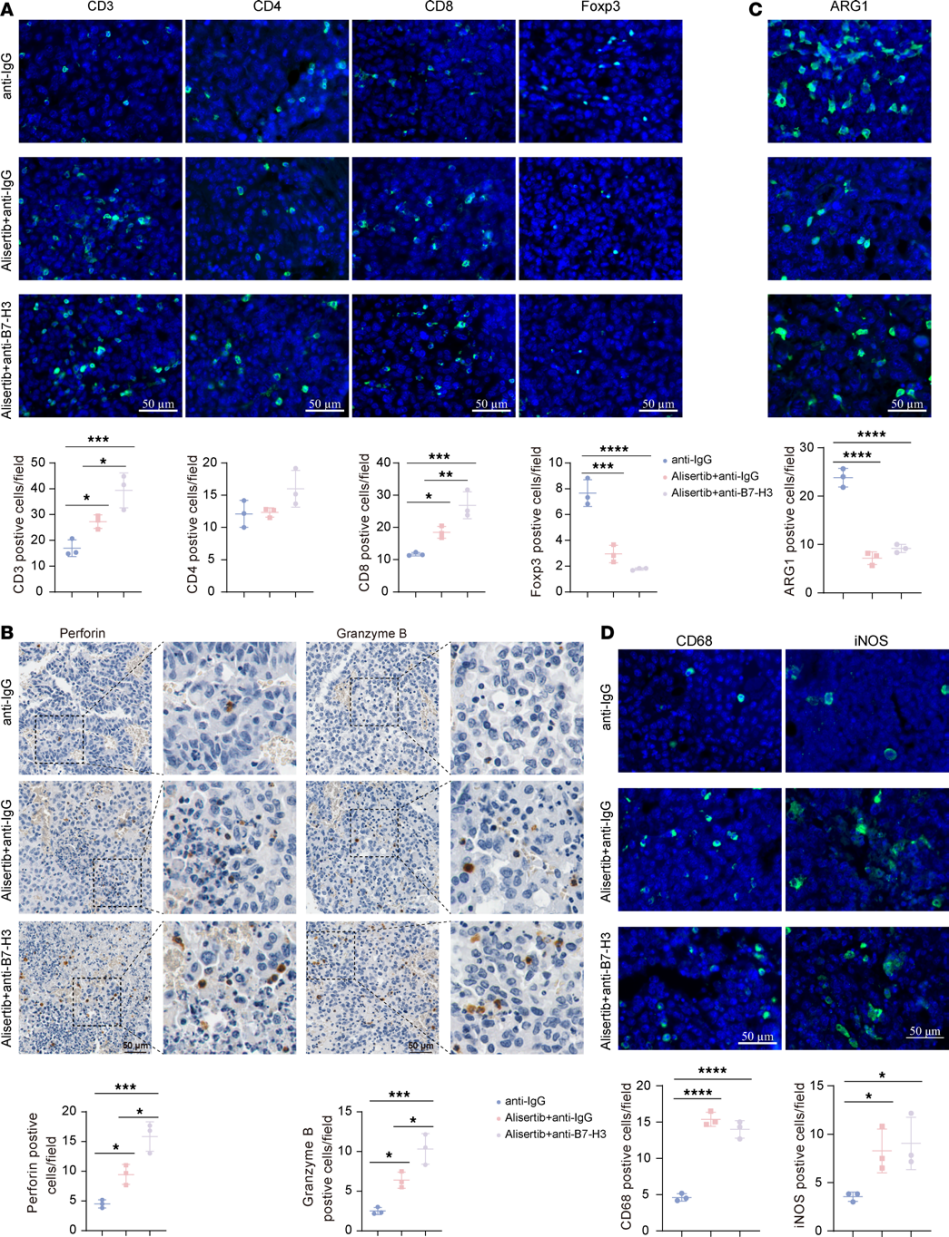
The combination of alisertib with an anti–B7-H3 mAb increases CD8^+^ T cell infiltration and activation. (**A**) A panel of immune markers in orthotopic G261 tumors from the anti-IgG, alisertib + anti-IgG, and alisertib + anti–B7-H3 groups were detected via immunofluorescence analysis, and the quantifications are shown on the below (*n* = 3/group). The T cell markers are CD3, CD4, CD8, and Foxp3. (**B**) Perforin and granzyme B in orthotopic G261 tumors from the anti-IgG, alisertib + anti-IgG, and alisertib + anti–B7-H3 groups were detected via IHC analysis, and the quantifications are shown on the below (*n* = 3/group). (**C** and **D**) A panel of immune markers in orthotopic G261 tumors from the anti-IgG, alisertib + anti-IgG, and alisertib + anti–B7-H3 groups were detected via immunofluorescence analysis, and the data are presented on the below (*n* = 3/group). The following markers of macrophages were used: CD68, iNOS, and ARG1. Statistical significance was assessed via 1-way ANOVA followed by Tukey’s multiple-comparison test (**A**–**D**). The data are presented as the mean ± SD (**A**–**D**). *P < 0.05, **P < 0.01, ***P < 0.001, ****P < 0.0001. Scale bars: 50 µm.

Since the AURKA inhibitor alisertib can upregulate B7-H3 expression and modulate the immune microenvironment in glioma cells, thereby increasing response rate and clinical efficacy of B7-H3–specific blocking mAbs, we hypothesized that combining an AURKA inhibitor with an anti–B7-H3 mAb could improve GBM patient prognosis. We created an orthotopic syngeneic model to investigate this hypothesis by injecting 2 × 10^5^ G261-Luc cells into the mouse frontal lobe. Twelve days after inoculation, the mice were randomized into 4 groups: (a) anti-IgG, (b) anti–B7-H3 mAbs, (c) alisertib and anti-IgG, and (d) alisertib and anti–B7-H3 mAbs. For groups c and d, alisertib was administered for 2 days before being combined with an i.p. injection of either an anti–B7-H3 mAb or an isotype control antibody (Figure 8A). Compared with alisertib or anti–B7-H3 mAb monotherapy, alisertib combined with anti–B7-H3 mAbs had a synergistic inhibitory effect on tumor growth (Figure 8, B–D, and Supplemental Figure 4D). On day 33 after inoculation with tumor cells, the tumors in the alisertib and anti–B7-H3 mAbs group were significantly smaller than those in the alisertib and anti-IgG group (Figure 8D). However, this result may be biased as it was measured on smaller tumors due to some mice dying from tumor burden. Moreover, our data reveal that the combination treatment of alisertib and anti–B7-H3 mAbs significantly extended animal survival compared with the anti-IgG (*P* = 0.0026), alisertib and anti-IgG (*P* = 0.0197), and anti–B7-H3 mAbs (*P* = 0.0090) groups, as demonstrated by Kaplan-Meier analysis (Figure 8E). Owing to the lack of significant difference in survival between anti-IgG and anti–B7-H3 mAbs, we analyzed tumor-infiltrating immune cells among (a) anti-IgG, (c) alisertib and anti-IgG, and (d) alisertib and anti–B7-H3 mAbs. Compared with combination therapy with alisertib and anti-IgG, combination therapy with alisertib and anti–B7-H3 mAbs significantly increased the abundance of total CD3^+^ T cells, tumor-infiltrating CD8^+^ T cells, perforin^+^ cells and granzyme B^+^ cells but not total CD4^+^ T cells (Figure 9, A and B). Additionally, the number of Foxp3^+^ T cells in the combination therapy with alisertib and anti–B7-H3 mAbs group was lower than that in the combination therapy with alisertib and anti-IgG group, but there was no statistically significant difference between the 2 groups (Figure 9A). We did not find a significant difference in the number of CD68^+^, iNOS^+^, or ARG1^+^ cells between the combination therapy with alisertib and anti–B7-H3 mAbs and the combination therapy with alisertib and anti-IgG groups (Figure 9, C and D), indicating that anti–B7-H3 mAbs treatment did not substantially alter the level of macrophage infiltration induced by alisertib treatment.

## Discussion

We present what we believe to be the first investigation into the potential of combining the AURKA inhibitor alisertib with B7-H3–specific blocking mAbs in preclinical glioma models. We discovered that alisertib upregulated EGFR/B7-H3 signaling and enhanced the innate immune response. Furthermore, combination therapy with alisertib and B7-H3–specific blocking mAbs resulted in increased infiltration of CD8^+^ cells into the tumor microenvironment, providing a potential treatment strategy for malignant GBM.

In humans, B7-H3 expression is limited to normal tissues. Nevertheless, it is aberrantly high in most cancer types (23, 24) and is strongly associated with poor prognosis and decreased overall survival in patients with cancer (4, 24–26). As an immune checkpoint, B7-H3 can inhibit T cell and NK cell activation in the tumor immune microenvironment, making it an attractive biomarker and target for immunotherapy. The experimental B7-H3 silencing and the development of B7-H3 blocking antibodies reduce cancer cell malignant potential (27). In recent years, progress has been made in clinical trials investigating B7-H3 (3, 24, 28). Many antibody-based strategies targeting B7-H3–expressing tumor cells have demonstrated potent antitumor activity and exhibited favorable safety profiles in preclinical models (3). However, there is a need to understand how the therapeutic efficacy of targeted B7-H3 immunotherapy can be enhanced.

The upregulation of inhibitory immune checkpoints has been associated with increased response rates and enhanced efficacy of antibody-based immunotherapies targeting these checkpoints (29, 30). Thus, inducing B7-H3 expression in GBM can increase cell response to anti–B7-H3 immunotherapy. Here, we found that B7-H3 expression was upregulated in glioma cells, which is consistent with the findings of previous studies (31, 32). Additionally, our data reveal a positive correlation between the expression of *AURKA* and *CD276* in GBM on the basis of the TCGA and CCGA databases. Furthermore, we confirmed in vitro that AURKA upregulation increased B7-H3 expression levels and that AURKA downregulation reduced B7-H3 expression levels in glioma cells.

In this study, we found that AURKA regulated B7-H3 expression through EGFR phosphorylation. Several studies have demonstrated the association between the upregulation of B7-H3 and EGFR signaling (20, 21). Previous studies have revealed that AURKA binds to SDCBP and phosphorylates it, inhibiting its ubiquitination-mediated degradation. Importantly, accumulated SDCBP activates EGFR by binding to it and preventing its internalization (16). Similarly, our findings revealed that AURKA modulates the EGFR/B7-H3 pathway, which may result from the maintenance of SDCBP. We observed that the knockdown of AURKA reduced SDCBP, EGFR phosphorylation at Tyr1068, and B7-H3 expression levels, whereas the administration of the EGFR activator NSC228155 or EGF restored EGFR phosphorylation and B7-H3 expression. Conversely, overexpression of AURKA increased SDCBP, EGFR phosphorylation at Tyr1068, and B7-H3 expression levels, and the changes in EGFR phosphorylation and B7-H3 expression were reversed by treatment with the EGFR inhibitor erlotinib. Although we did not investigate the effect of SDCBP knockdown or KO on expression of EGFR phosphorylation, based on our findings and previous studies (16), we speculated that there are potential interactions between AURKA, SDCBP, and EGFR in regulating B7-H3. The AURKA has been reported to interact with numerous proteins that play crucial roles in carcinogenesis (33, 34). However, the exact mechanism underlying the AURKA-mediated activation of EGFR requires further investigation.

After treatment with alisertib, an AURKA-specific inhibitor, we observed a marked reduction in the proliferation of glioma cells, accompanied by an increase in B7-H3 expression. Our data in vivo show that alisertib treatment reduced intracranial tumor volume and prolonged mouse survival. Alisertib has exhibited considerable antitumor effects in preclinical studies and is the only AURKA inhibitor in phase III clinical evaluation (10, 11, 35–37). Intriguingly, alisertib not only fails to decrease B7-H3 expression but instead induces its upregulation. Given that elevated levels of B7-H3 can potentiate tumor cell response to targeted B7-H3 therapy, a promising strategy for treating GBM could involve combining AURKA inhibitors (such as alisertib) with monoclonal antibodies targeting B7-H3 (27, 28).

Furthermore, we showed that alisertib treatment increased B7-H3 expression through EGFR phosphorylation. The mechanism by which alisertib contributes to EGFR-Tyr1068 phosphorylation is still unknown. Previous studies demonstrated that the AURKA inhibitor VE-465 suppressed the phosphorylation of AURKA-T288 and EGFR-Thr654 and -Ser1046 but not the phosphorylation of EGFR-Tyr1068. These results also suggested that the phosphorylation of EGFR at Tyr1068 could not be regulated by AURKA kinase activity (38). Thus, we propose that the increased expression of total AURKA protein by alisertib prevents the degradation of SDCBP, which increases EGFR phosphorylation and leads to the upregulation of B7-H3 expression. However, the increased EGFR phosphorylation caused by alisertib has the potential to increase the sensitivity of GBM to EGFR inhibitors. In some types of lung cancer, AURKA inhibitors in combination with EGFR inhibitors have been shown to provide greater tumor suppression than monotherapy (39).

Subsequently, we found that alisertib treatment selectively upregulated the expression of B7-H3 in glioma cells but not in normal astrocytes, which has not been previously reported, to our knowledge. Our data demonstrate that the administration of alisertib induced the upregulation of B7-H3 expression in various glioma cell lines and the 293T cell line, leaving normal astrocytes unaffected. Consistent with previous studies, our findings also suggest that EGFR signaling might regulate AURKA expression at the transcriptional level, as evidenced by the upregulation of AURKA expression after treatment with EGF (39, 40). Additionally, we conducted in vitro experiments to confirm the effects of alisertib on B7-H3 expression in mouse glioma cells. Our data demonstrate that alisertib administration induced B7-H3 expression in G261 cells, further confirming our hypothesis.

In this study, we found that alisertib treatment not only induced B7-H3 expression in glioma cell but also improved the immune microenvironment in mouse models, potentially increasing the response of tumors to anti–B7-H3 immunotherapy (41). The immunosuppressive microenvironment is considered a critical contributor to resistance in anti–B7-H3 immunotherapy. Our data demonstrate that alisertib treatment reduced the number of tumor-promoting immune cells, including M2 macrophages and Tregs, and increased the number of antitumor immune cells, including M1 macrophages and CD8^+^ T cells (42, 43). Although the effect of alisertib on the immune microenvironment is not fully understood (12), these responses of the immune microenvironment to AURKA inhibition may contribute to the suppression of tumor progression and increased responsiveness to anti–B7-H3 therapy. Previous studies have shown that alisertib eliminates myeloid cell–mediated immunosuppression in breast cancer and inhibits the accumulation of tumor-promoting myeloid cells, including myeloid-derived suppressor cells, in various tumor models (12), suggesting that these mechanisms of action may also play a role in the therapeutic efficacy of alisertib in GBM tumor.

Compared with treatment with either alisertib or anti–B7-H3 alone, the combination treatment markedly prolonged survival time. However, cerebral edema was also evident during the combination treatment and in the group treated with anti–B7-H3, which may be related to the immune response (44, 45). Moreover, our data reveal significant differences in cytotoxic CD8^+^ T cell infiltration and activation between the combination group and the group treated with alisertib alone, while there was no significant difference in the infiltration of CD4^+^ T cells and myeloid cells. It remains uncertain whether the upregulation of B7-H3 induced by alisertib is responsible for these microenvironmental changes. To address this issue, one way is to conduct similar experiments using GBM cell lines with B7-H3 gene KO.

However, our study has limitations. Firstly, in this study, we employed immunofluorescence to assess the effect of combination therapy on the immune microenvironment. Given the limited size of mouse intracranial tumor tissue, it presents a challenge to observe the infiltration of immune cells in the tumor microenvironment simultaneously using immunofluorescence and flow cytometry. Additionally, due to difficulties in constructing an immune-resistant syngeneic model with mouse CT-2A cells or a patient-derived tumor xenograft (PDX) model, we utilized a syngeneic mouse model with G261 cells to investigate changes of immune cells infiltration in the immune microenvironment induced by combination treatment. Therefore, future research should consider employing more rigorous preclinical models, such as a PDX model and a syngeneic model that possesses low mutational burden, which is known to exhibit resistance to immunotherapy-induced tumor cell killing.

In conclusion, our study proposes an AURKA-mediated EGFR/B7-H3 regulatory mechanism in glioma cells and highlights the importance of AURKA in enhancing B7-H3–targeting immunotherapy in glioma mouse models. Our findings suggest that combining alisertib with B7-H3–specific blocking mAbs may be an effective therapy for treating GBM and other cancers.

## Methods

### Sex as a biological variable.

Sex was not considered as a biological variable. Both U87-MG cells and LN18 cells were derived from male patients, while LN229 cells originate from a female patient. The 2 patient-derived GBM cell lines, LHG and LS, were obtained from male patients. For in vivo studies, only female mice were used based on practical considerations.

### RNA-Seq dataset and analysis.

The RNA-Seq dataset and the corresponding clinical information of LGG and patients with GBM were obtained from CGGA database (http://www.cgga.org.cn/) and TCGA (the UCSC Xena browser; https://gdc.xenahubs.net). Raw data from the CGGA or the TCGA were displayed as fragments per kilobase of exon model per million mapped fragments (FPKM). The FPKM value was subsequently converted into a transcript per kilobase million value. The CGGA cohort was used as the experimental set, and the TCGA cohort was used as the verification set.

The cell cycle gene set (Supplemental Table 1) was obtained from the genes annotated under the cell cycle GO term (accession no. GO:0007049), and the immune checkpoint gene set (Supplemental Table 2) was downloaded from the Sinobiological database (https://www.sinobiological.com/category/immunocheckpoint-proteins-list).

Differential expression gene set analysis on the Sangerbox platform (http://sangerbox.com/) was employed to identify the DEGs between WHO II-IDH mutation gliomas and WHO IV-IDH WT gliomas. We accepted the criteria of |log_2_(fold change)| > 1 and *P* < 0.01 to identify the DEGs in the CGGA and TCGA datasets, respectively. Next, we identified the cell cycle and immune checkpoint genes among the DEGs identified in both the TCGA and CGGA databases via the intersection format based on the cell cycle gene set and the immune checkpoint gene set described above for further analysis. The correlation between the selected cell cycle genes and immune checkpoint genes was determined via Spearman correlation analysis using the R package “correlation,” and a heatmap of the correlation results was drawn with the R package “ggplot2.” Differential expression of *AURKA* and *CD276* between GBM and normal brain tissues was analyzed in GEPIA2 (http://gepia2.cancer-pku.cn/#analysis) via matched TCGA normal and GTEx data.

### Samples and reagents.

To validate AURKA and B7-H3 expression in gliomas of different grades, GBM (WHO IV, *n* = 4), LGG (WHO II, *n* = 4), and peritumoral (normal tissue, *n* = 4), surgical specimens were obtained from Beijing Tiantan Hospital for Western blotting. Detailed patient information is presented in Supplemental Table 3.

Alisertib (MLN8237) (S1133), erlotinib (S7786), and NSC288155 (S8312) were acquired from Selleck. EGF (HY-P7109) was purchased from MedChemExpress.

### Cell culture.

The human glioma cell lines LN18, U87-MG, and LN229; the human embryonic kidney cell line 293T; and the mouse glioma cell line G261 were cultured in DMEM (Thermo Fisher Scientific, C11995500BT) supplemented with 10% FBS (Thermo Fisher Scientific, 10099-141) and 1% glutamine (Thermo Fisher Scientific, 25030081) in an incubator at 37°C with 5% CO_2_. NHA cells (ScienCell, 1800) were cultured in astrocyte medium (ScienCell, 1801) which contains 5% FBS, astrocyte growth supplement, and 1% penicillin/streptomycin in an incubator at 37°C with 5% CO_2_. The 2 patient-derived GBM cell lines, LHG and LS, were previously established and cultured in DMEM, B27 (Scintol, S6015, 1:100), N2 (Scintol, S6017, 1:200), insulin (Scintol, SC25800, 10 μg/mL), bFGF (Scintol, SC107, 10 ng/mL), EGF (Scintol, sC102, 10 ng/mL), and 5% FBS in an incubator at 37°C with 5% CO_2_. LN18, U87-MG, LN229, 293T, and G261 cells were purchased from the American Type Culture Collection. The cell lines used in this study were tested for mycoplasma contamination via the Mycoplasma Detector and tested negative (Vazyme, D101-02). The respective cell line sources authenticated the cells.

### Quantitative PCR (qPCR) assay.

Total RNA was isolated via a RNeasy Mini Kit (QIAGEN, 74104) and reverse transcribed to cDNA via an S6 Super qPCR RT Kit (Science Tool, S6166). The cDNA was amplified via Power SYBR Green (Applied Biosystems, 4367659) by Quant Studio 5 (Applied Biosystems). The amplification program was as follows: initial denaturation step at 95°C for 30 seconds, followed by 40 cycles at 95°C for 15 seconds and 60°C for 60 seconds. All the assays were performed in triplicate, and the average fold changes were calculated on the basis of mRNA *ACTB* in the threshold cycle. The relative quantitative value for each gene was determined by via the 2^−ΔΔCT^ method. The primer sequences are listed in Supplemental Table 4.

### Western blotting analysis.

The samples were lysed with nondenaturing lysis buffer (Applygen, C1050) supplemented with 1% protease inhibitor cocktail (Solarbio, P6730) and 1% phosphatase inhibitor (Solarbio, P1260). The samples subjected to new flash protein AnyKD PAGE (Dakewe, 8012011) and transferred to polyvinylidene fluoride (PVDF) membranes (Merck Millipore Ltd., IPVH00010). The membrane was subsequently blocked with 5% nonfat milk in TBST (0.1% Tween 20) and probed with primary target antibodies and corresponding secondary antibodies conjugated to horseradish peroxidase. The following primary antibodies were used: rabbit anti–Aurora A (CST, 14475; 1:2,000), rabbit anti–Aurora A (Abcam108353; 1:2,000), rabbit anti–p-Aurora A (T288) (CST, 2914; 1:2,000), rabbit anti-EGFR (CST, 2232; 1:2,000), rabbit anti–p-EGFR (Tyr1068) (CST, 3777; 1:1,000), rabbit anti–B7-H3 (CST, 14058; 1:3,000), rabbit anti–B7-H3 (Abcam134161; 1:3,000), rabbit anti-SDCBP (Abcam19903; 1:3,000), and mouse anti–β-actin (Boao Ruijing Technology Development Co., Ab1015t; 1:5,000) conjugated to horseradish peroxidase. The following secondary antibody was used: anti–rabbit IgG secondary antibody and HRP (Abcam, ab6721; 1:5,000). The specific protein bands were visualized via enhanced chemiluminescence reagents (NCM Biotech, P10300) on an Amersham Imager 600 (GE). All the assays were performed in triplicate. The final data were subjected to grayscale scanning and semiquantitative analysis via ImageJ software (NIH; https://imagej.net/ij/download.html).

### Flow cytometric analysis.

The cells were harvested with 0.25% trypsin, washed with cell staining buffer (BioLegend, 420201), and incubated with Human TruStain FcX (BioLegend, 422301) at room temperature. The cells were subsequently incubated with anti–B7-H3 (BioLegend, 351004 or 351006) or isotype control antibodies (BioLegend, 400112 or 400322) at 4°C for 15 minutes. Signals were detected on a flow cytometer (BD Accuri C6 Plus), and the data were analyzed with FlowJo software version 10.6 (Tree Star Inc.). The cells were gated through forward scatter (FSC) and side scatter (SSC) and then analyzed for the corresponding fluorescence of B7-H3. All the assays were performed in triplicate.

### Lentiviral infection and small interfering RNA transfection.

Lentiviral plasmids carrying *AURKA*-cDNA-Flag or shRNAs targeting *AURKA* were used to upregulate or knock down the expression of AURKA, respectively. Lentiviral plasmids carrying *AURKA*-cDNA-Flag or shRNAs targeting *AURKA* were purchased from Beijing Yibaike Biotechnology Co. Ltd. and Tsingke Biotechnology Co. Ltd. The shRNA sequences targeting *AURKA* are listed in Supplemental Table 5.

Small interfering RNAs (siRNAs) targeting *AURKA* were used to knock down the expression of AURKA with Lipofectamine RNAiMAX (Invitrogen, 13778075) according to the manufacturers’ instructions. siRNAs targeting *AURKA* were purchased from Genomeditech Co. Ltd. The sequences of the siRNAs targeting *AURKA* are listed in Supplemental Table 6.

### Cell viability assay.

Cell viability was assessed by a Cell Counting Kit-8 (CCK-8) assay (Biosharp, BS350A). Ninety-six–well plates were seeded with approximately 4 × 10^3^ cells per well. The cells were allowed to attach overnight. The cells were subsequently treated with various concentrations of alisertib. Cell viability was evaluated with a CCK-8 assay. The absorbance values were determined at 450 nm on a Synergy H1 MFD spectrophotometer (BioTek). All the assays were performed in at least triplicate.

### Orthotopic syngeneic mouse model experiments.

C57BL/6 mice (Beijing Vital River Laboratory Animal Technology Co. Ltd.) were anesthetized with isoflurane and fixed in a stereotactic head frame, and a burr hole was drilled on the coronal suture 2.0 mm lateral (right) to the bregma. G261 cells (200,000 cells in 5 μL of PBS) expressing luciferase by lentiviral infection (G261-Luc) were slowly injected into the brain at a depth of 3.5 mm via a Hamilton syringe. Bone wax was used to close the hole, and the wound was sutured. Twelve days after surgery, the C57BL/6 mice were imaged via an IVIS (PerkinElmer) 10 minutes after 150 mg/kg D-luciferin (PerkinElmer, 122799) was i.p. injected. The fluorescence intensity was quantified via Living Image, a software program provided by the same manufacturer. The mice were imaged every 7 days via the IVIS system to evaluate tumor volume.

### Orthotopic xenograft mouse models.

NSG mice (Beijing SPF Biotechnology Co. Ltd.) were anesthetized with isoflurane and fixed in a stereotactic head frame, and a burr hole was drilled on the coronal suture 2.0 mm lateral (right) to the bregma. U87-MG cells (200,000 cells in 5 μL of PBS) expressing luciferase via lentiviral infection (U87-MG–*Luc*) were slowly injected into the brain at a depth of 3.5 mm via a Hamilton syringe. Bone wax was used to close the hole, and the wound was sutured. Twelve days after surgery, the NSG mice were imaged via IVIS 10 minutes after 150 mg/kg D-luciferin was i.p. injected. The fluorescence intensity was quantified via Living Image, a software program provided by the same manufacturer. The mice were imaged every 7 days via the IVIS system to evaluate tumor volume.

### Administration of alisertib and anti–B7-H3 mAbs in vivo.

Alisertib was suspended in 10% 2-hydroxypropyl-b-cyclodextrin (MilliporeSigma, H107) and 1% sodium bicarbonate (MilliporeSigma, S5716) in water. When tumor growth was measurable (~12 days after the injection), the C57BL/6 mice were assigned to 2 groups that received the vehicle orally (100 μL of 10% 2-hydroxypropyl–cyclodextrin and 1% sodium bicarbonate) or alisertib (30 mg/kg in a final formulation in 10% 2-hydroxypropyl–cyclodextrin/1% sodium bicarbonate) for 14 consecutive days. For combination therapy, alisertib was administered for 2 days before combination with an i.p. injection of either anti–B7-H3 mAbs (300 μg every 2 days, a total of 4 times; BE01224 clone MJ18, BioXcell) or an isotype control antibody (300 μg every 2 days, a total of 4 times; BE0088, clone HRPN, BioXcell).

### IHC.

The tumor samples were fixed in 10% buffered formalin and embedded in paraffin. The sections cut from the paraffin-embedded blocks were deparaffinized with xylene and ethanol. The samples were subsequently heated by microwaving in 10 mM Na citrate buffer or EDTA antigen retrieval solution for 20 minutes for antigen retrieval. The endogenous peroxidase of the sections was subsequently inactivated, and the sections were blocked at room temperature for 1 hour with goat serum. The sections were incubated overnight at 4°C with primary target antibodies. The following primary antibodies were used: anti–B7-H3 (CST, 14058), anti-perforin (CST, 31647), and anti-granzyme B (CST, 46890). Then, a secondary antibody solution (Abcam, ab6721; 1:5,000) was added at room temperature for 1 hour. The final signal was developed via a DAB Horseradish Peroxidase Color Development Kit (Lablead, D2003). IHC staining was assessed by combining intensity, which was scored as negative (score, 0), weak (score, 1+), moderate (score, 2+), strong (score, 3+) ,or extremely strong (score, 4+), with the extent of immunopositivity (percentage of positive cells). For B7-H3, the immunohistochemistry H-score = 1 × (percentage of 1+ cores) + 2 × (percentage of 2+ cores) + 3 × (percentage of 3+ cores) + 4× (percentage of 4+ cores).

### Immunofluorescence.

Immunofluorescence assays were performed on consecutive sections from syngeneic tumors removed from C57BL/6 mice. The tumor samples were fixed in 10% buffered formalin and embedded in paraffin. The sections cut from the paraffin-embedded blocks were deparaffinized with xylene and ethanol. The samples were subsequently heated by microwaving in 10 mM Na citrate buffer or EDTA antigen retrieval solution for 20 minutes for antigen retrieval. After rinsing in PBS, the sections were incubated overnight at 4°C with primary target antibodies. The following primary antibodies were used: anti-CD3 (Abcam, ab16669), anti-CD4 (Abcam, ab183685), anti-CD8 (Abcam, ab209775), anti-Foxp3 (Abcam, ab215206), anti-CD68 (Abcam, ab125212), anti-iNOS (Abcam, ab15323), and anti–Arginase 1 (Santa Cruz Biotechnology Inc., sc-271430) antibodies. After washing with PBS for 5 minutes 3 times, a secondary antibody solution (Zsbio, ZF-0311 or ZF-0312) was added at room temperature for 1 hour, followed by washing with PBS 3 times. Then, the DAPI solution (ZSGB-BIO, ZLI-9557) was applied, and the cover glass was mounted. Immunofluorescence was imaged with an Axio Observer.ZI microscope and quantification. All the assays were performed in triplicate.

### Protein preparation and proteomics analysis.

Protein preparation and proteomics analysis were completed by label-free proteomics quantitates (46–48). The cells in each group were lysed with nondenaturing lysis buffer (Applygen, C1050) supplemented with 1% protease inhibitor cocktail (Solarbio, P6730). The total protein concentration was measured with a bicinchoninic acid (BCA) kit (23227, Pierce). A mass of 100 μg of protein from cells was denatured, reduced, alkylated, and digested overnight with 0.1 μg/μL trypsin solution at 37°C. The peptides were injected onto a desalting column (ChromXP C18CL 5 μm 120 Å, 300 μm i.d.) and separated on an analytical column (ChromXP C18 3 μm, 120 Å, 300 μm i.d.) via an Eksigent nanoLC instrument (Eksigent). The samples separated via capillary high-performance liquid chromatography were subsequently analyzed via a Triple TOF 5600^+^ system (AbSciex). The identification of peptides and proteins was carried out via Protein Pilot software (version 5.0.2; Sciex) with a human Swiss-Prot database (2020). The tandem mass spectrometry (MS/MS) spectra of the peptides were subsequently used to create the spectral library for SWATH (sequential window acquisition of all theoretical fragment ion spectra) peak extraction via PeakView software (2.2). Peptides with confidence intervals greater than 99% were added to the spectral library. Six cell samples (shNC and sh*AURKA*#3 groups, triplicate each group) were evaluated via an data-independent acquisition (DIA) method. On the basis of the spectral library constructed, the SWATH-MS acquisition method was used. The SWATH method comprises TOF MS followed by 70 windows of variable size (350–1250 *m/z*, with an acquisition time of 90 ms). The SWATH variable window calculator from Sciex was used to adjust the window width of these variables to the ion density. Data extraction from SWATH runs was carried out via PeakView (2.2). Chromatograms of the extracted ions were created for each selected ionic fragment. PeakView calculates a score and FDR for each assigned peptide via chromatographic and spectral components. MarkerView (version 1.3.1; Sciex) allowed total area sum normalization, and 1-tailed Student’s *t* test was applied to test differential abundance.

### Statistics.

The Bliss independence model was employed to assess the synergistic effects of anti–B7-H3 mAbs and alisertib in mice (49). All the statistical values were calculated with GraphPad Prism 9.0 (GraphPad Software, Inc.). One-way ANOVA was used for comparisons between more than 2 groups, and Tukey’s multiple-comparison test was used for multiple comparisons. Student’s *t* tests were performed to compare the differences between 2 groups. The quantitative data are presented as the mean ± SD and the mean ± SEM from at least 3 samples or experiments per data point. A *P* value less than 0.05 was considered statistically significant.

### Study approval.

The study was approved by the Research Ethics Committee of Beijing Tiantan Hospital (no. KY2014-021-02), and informed consent was obtained from all enrolled subjects. The study was carried out in full compliance with all principles of the World Medical Association Declaration of Helsinki. All the animal protocols were approved by the Animal Welfare Ethics Committee of Beijing Neurosurgical Institute.

### Data availability.

The datasets used and analyzed during the current study, including Supporting Data Values for all figures, are available in the supplemental materials or from the corresponding author upon reasonable request.

## Author contributions

JF conceived the idea. NJ, Hong Wan, SH, YM, ZH, JW, SZ, HL, and YL collected the samples and performed part of the in vitro experiments. JF and ZP collected the RNA-Seq datasets and performed the bioinformatics analysis. JF, JL, YD, and BH established the cell model and performed the in vitro experiments. ZH, JW, TS, and NJ established the patient-derived GBM cells. SH, YD, and JL established the mouse model and performed the in vivo experiments. JF, SH, NJ, and ZZ interpreted the data. JF, Herui Wang, JL, and YD aided in the data analysis and wrote the manuscript. The number of experiments performed by each researcher was used to determine the order of the 2 co–first authors. All authors approved the submission.

## Supplementary Material

Supplemental data

Unedited blot and gel images

Supplemental tables 1-6

Supporting data values

## Version 1 02/10/2025

In-Press Preview

## Version 2 03/10/2025

Electronic publication
