# Supplementary material for: The AURKA inhibitor alters the immune microenvironment and enhances targeting B7-H3 immunotherapy in glioblastoma
Source: JCI Insight. 2025 Feb 10;10(5):e173700. doi: 10.1172/jci.insight.173700 (PMC11949004; doi:10.1172/jci.insight.173700)
Supplement: Supplemental data [file jciinsight-10-173700-s122.pdf]

Supplementary Figure 1

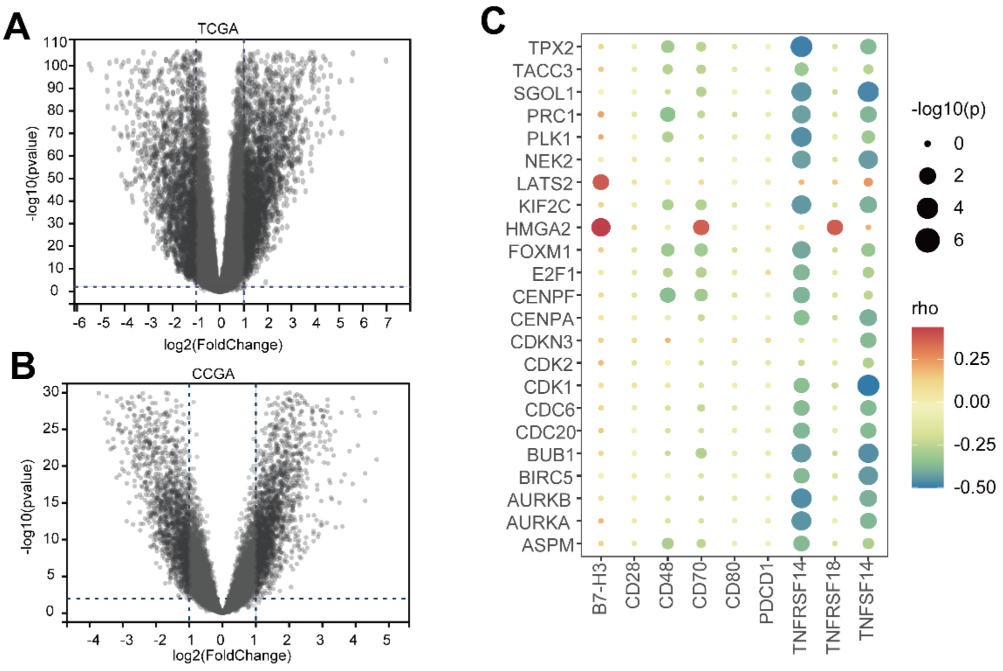

Supplementary Figure 1 (A and B) Volcano plots of the DEGs between GBM (WHO-IV and IDH wild type) and LGG (WHO-II and IDH mutant) based on the TGGA **(A)** and CGGA **(B)** databases. **(C)** Heatmaps displaying the correlations in the TCGA datasets between cell cycle genes among the DEGs in both the TCGA and CGGA cohorts and immune checkpoint genes among the DEGs in both the TCGA and CGGA cohorts.

Supplementary Figure 2

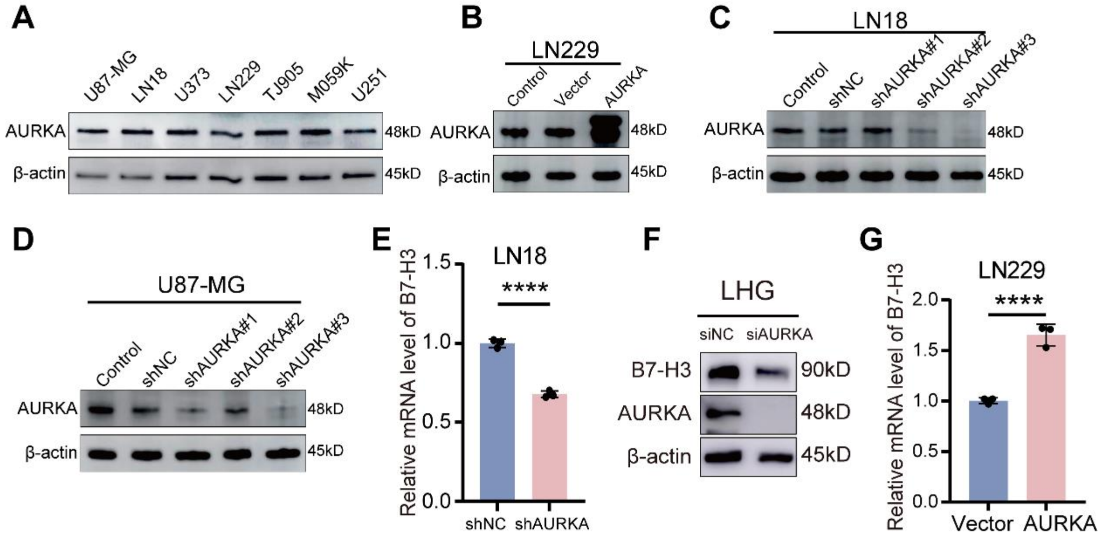

Supplementary Figure 2 (A) The protein expression of AURKA in the human glioma cell lines U87-MG, LN18, U373, LN229, TJ905, M059K and U251 was detected by Western blotting. β-actin was used as the internal control. **(B)** Overexpression of AURKA by AURKA_cDNA_Flag in LN229 cells. The protein expression of AURKA was detected via Western blotting. β-actin was used as the internal control. **(C and D)** Silencing of AURKA with specific shRNAs in LN18 and U87-MG cells. The protein expression of AURKA was detected via Western blotting. β-actin was used as the internal control. **(E)** The mRNA level of B7-H3 in LN18 cells expressing shNC or AURKA-shRNA#3. **(F)** The protein expression of AURKA and B7-H3 in LHG cells transfected with AURKA-siRNA or siNC was detected by Western blotting. β-actin was used as the internal control. **(G)** The mRNA level of B7-H3 in LN229 cells expressing vector or AURKA_cDNA_Flag. Statistical significance was assessed by a 2-tailed unpaired Student's t test and the data represent the means ± SD **(E**

and G). All samples were biologically independent, and 3 independent experiments were performed. ****P <0.0001.

Supplementary Figure 3

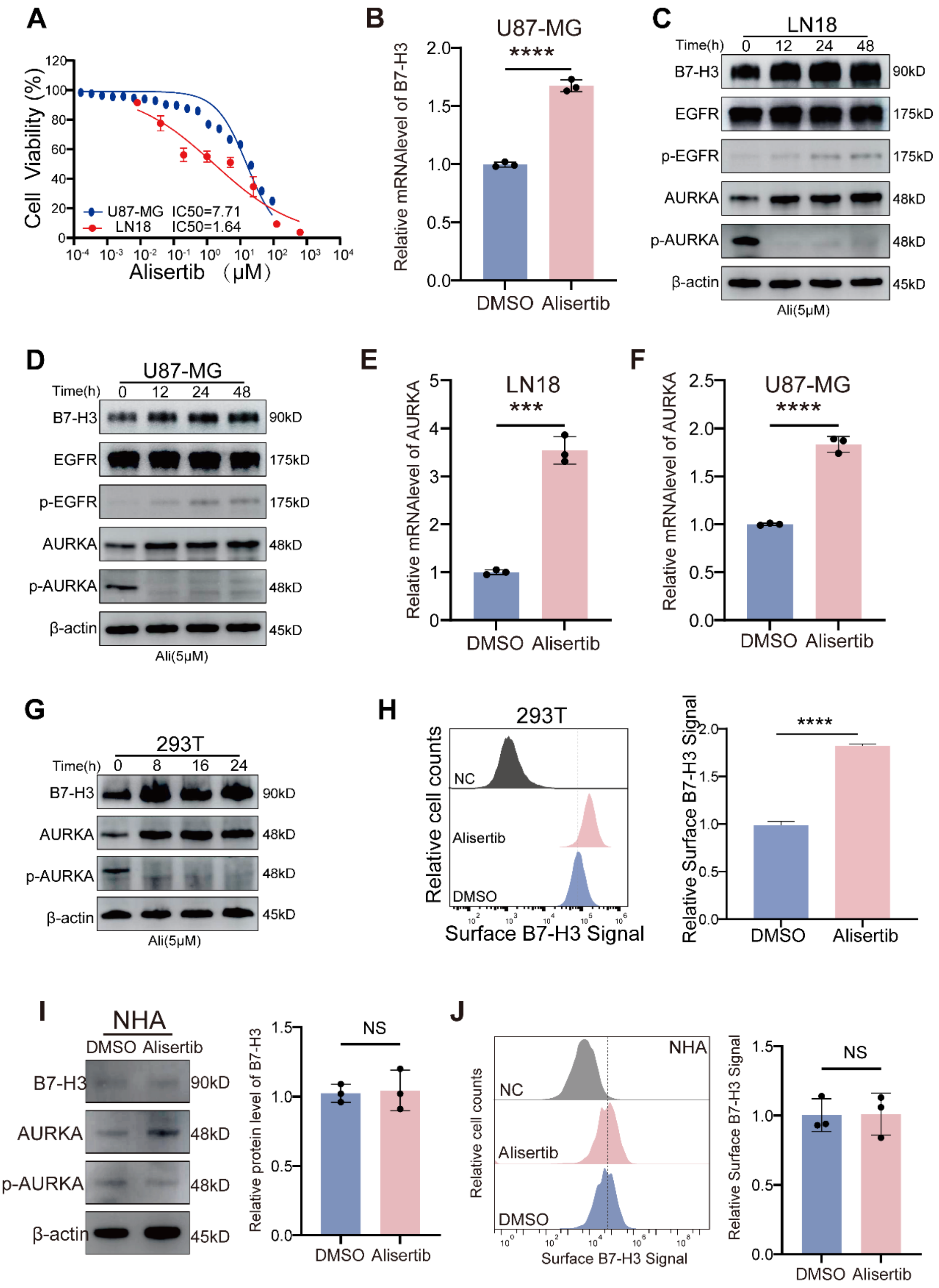

Supplementary Figure 3 (A) LN18 (n = 6 independent samples) and U87-MG (n=3 independent samples) cells were treated with increasing concentrations of alisertib for 72 h. The cell viability was analyzed by a

CCK-8 assay. **(B)** The mRNA level of B7-H3 in U87-MG cells treated with alisertib (24 h, 5 μM) or DMSO. **(C and D)** The protein expression of B7-H3, total AURKA, p-AURKA (T288), p-EGFR (Y1068) and total EGFR in LN18 and U87-MG cells treated with alisertib (5 μM) for different durations (0 h, 12 h, 24 h and 48 h) was detected by Western blotting. β-actin was used as the internal control. **(E and F)** mRNA level of AURKA in LN18 and U87-MG cells treated with alisertib (24 h, 5 μM) or DMSO. **(G)** The protein expression of B7-H3, total AURKA and p-AURKA (T288) in 293T cells treated with alisertib (5 μM) for different durations (0 h, 8 h, 16 h and 24 h) was detected via Western blotting. β-actin was used as the internal control. **(H)** B7-H3 levels on the cell surface of 293T cells treated with alisertib (24 h, 5 μM) or DMSO were quantified via flow cytometry. Cells that were only stained with isotype control antibodies were used as the negative control (NC). (I) The protein expression of B7-H3, total AURKA and p-AURKA (T288) in NHA cells treated with alisertib (24 h, 5 μM) or DMSO was detected by Western blotting. β-actin was used as the internal control. **(J)** B7-H3 levels on the cell surface of NHA cells treated with alisertib (24 h, 5 μM) or DMSO were quantified via flow cytometry. Cells that were only stained with isotype control antibodies were used as the negative control (NC). Statistical significance was assessed by using a 2-tailed unpaired Student's t test and the data represent the means ± SD (B, E, F, H, I and J). All the samples were biologically independent. Three

independent experiments were performed **(B-J).** ***P <0.001, ****P <0.0001. NS, not significant.

Supplementary Figure 4

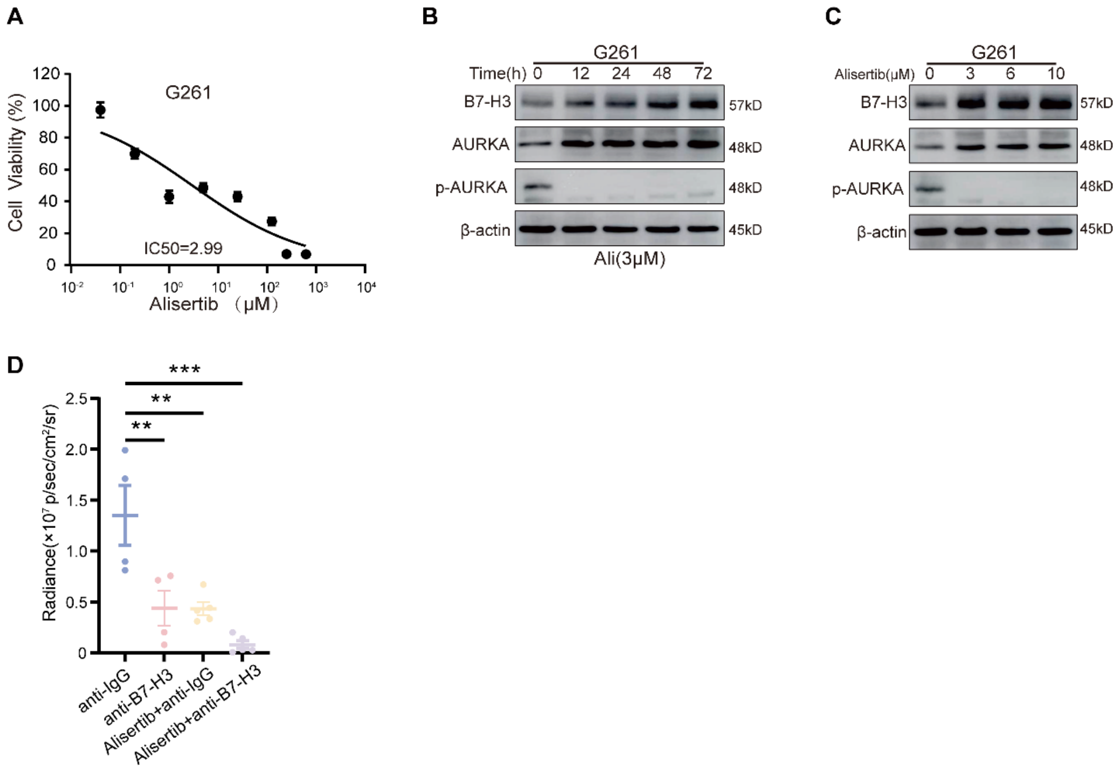

Supplementary Figure 4 (A) G261 cells were treated with increasing concentrations of alisertib for 72 h, and cell viability was analyzed via a CCK-8 assay. (n = 6 independent samples). **(B)** The protein expression of B7-H3, total AURKA, and p-AURKA (T288) in G261 cells treated with alisertib (3 μM) for different periods (0 h, 12 h, 24 h, 48 h or 72 h) was detected by Western blotting (n = 3/group). β-actin was used as the internal control. **(C)** The protein expression of B7-H3, total AURKA, and p-AURKA (T288) in G261 cells treated with increasing concentrations of alisertib for 24 h was detected by Western blotting (n = 3/group). β-actin was used as the internal control. **(D)** On day 26, the tumor volume in the alisertib + anti-IgG (n = 5), anti-B7-H3 mAb (n = 4), and combination treatment (n = 5) groups was significantly lower than that in the anti-IgG

group (n = 4). The tumors in the alisertib + anti-B7-H3 mAb group were smaller than those in the alisertib + anti-IgG or anti-B7-H3 mAb group, but the difference was not statistically significant. Statistical significance was assessed via 1-way ANOVA followed by Tukey's multiple comparisons test and the data represent the means ± SEM (D). **$P<0.01$, ***$P<0.001$.
