# Supplementary material for: The AURKA inhibitor alters the immune microenvironment and enhances targeting B7-H3 immunotherapy in glioblastoma
Source: JCI Insight. 2025 Feb 10;10(5):e173700. doi: 10.1172/jci.insight.173700 (PMC11949004; doi:10.1172/jci.insight.173700)

Figure 1J

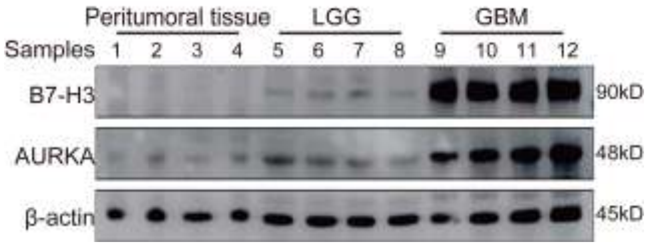

unedited gel

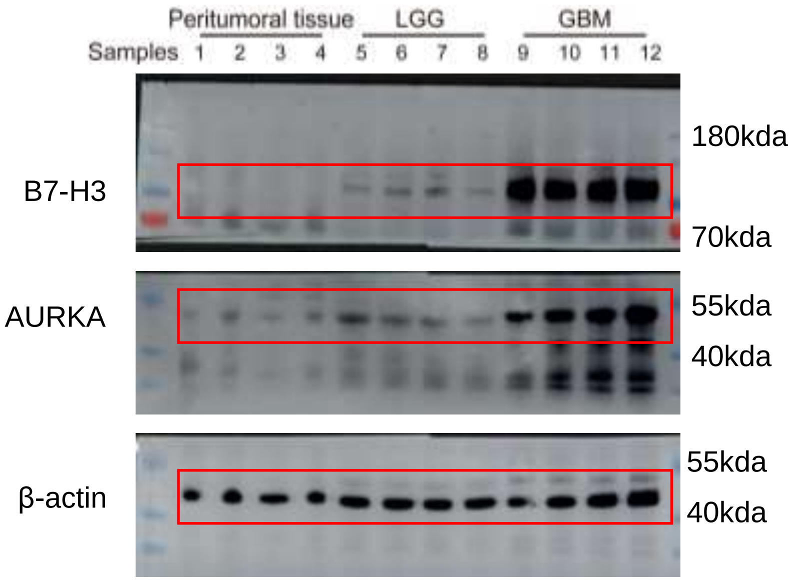

Figure 2B

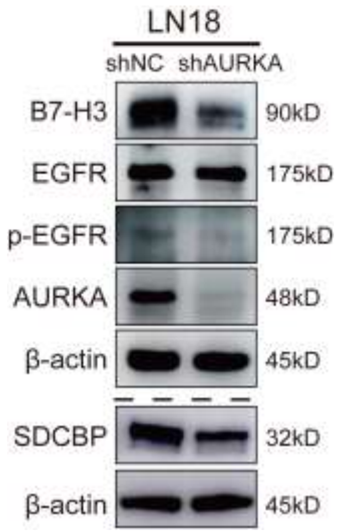

unedited gel

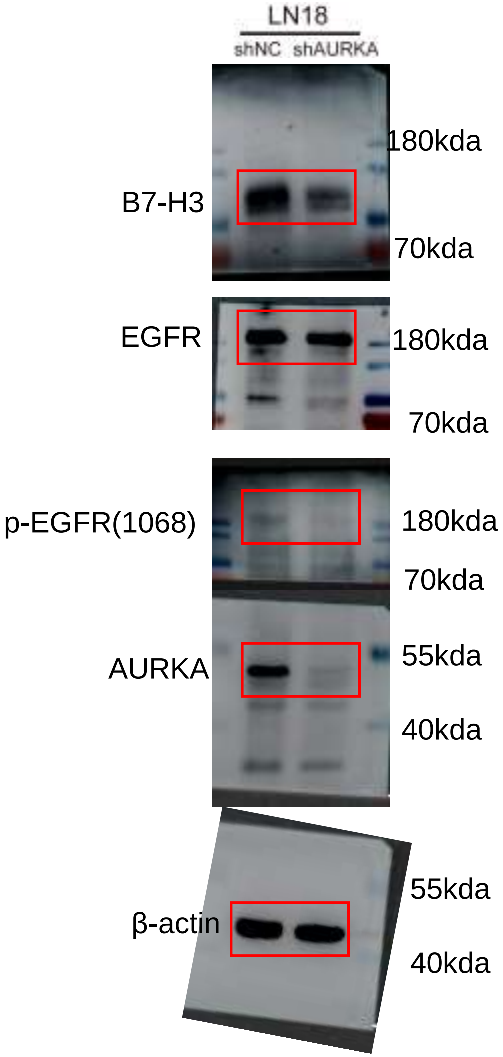

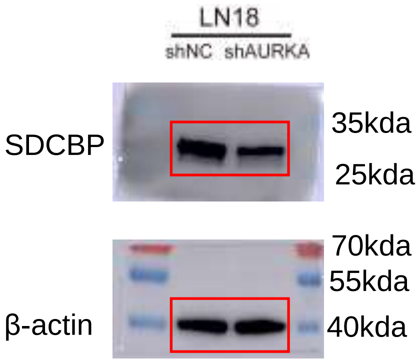

Figure 2C

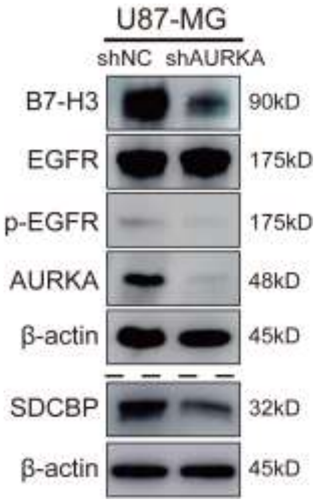

unedited gel

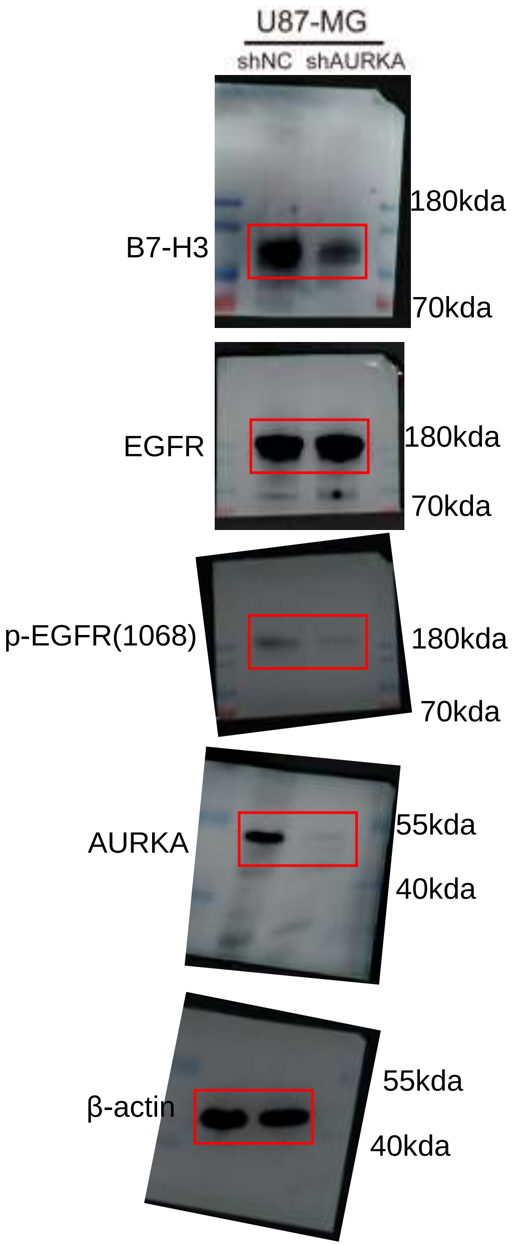

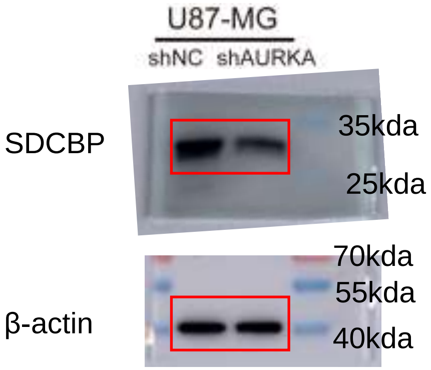

Figure 2E

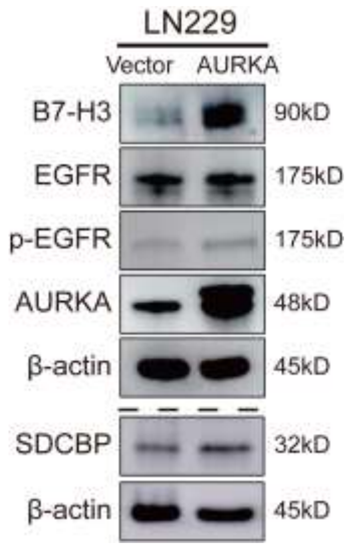

unedited gel

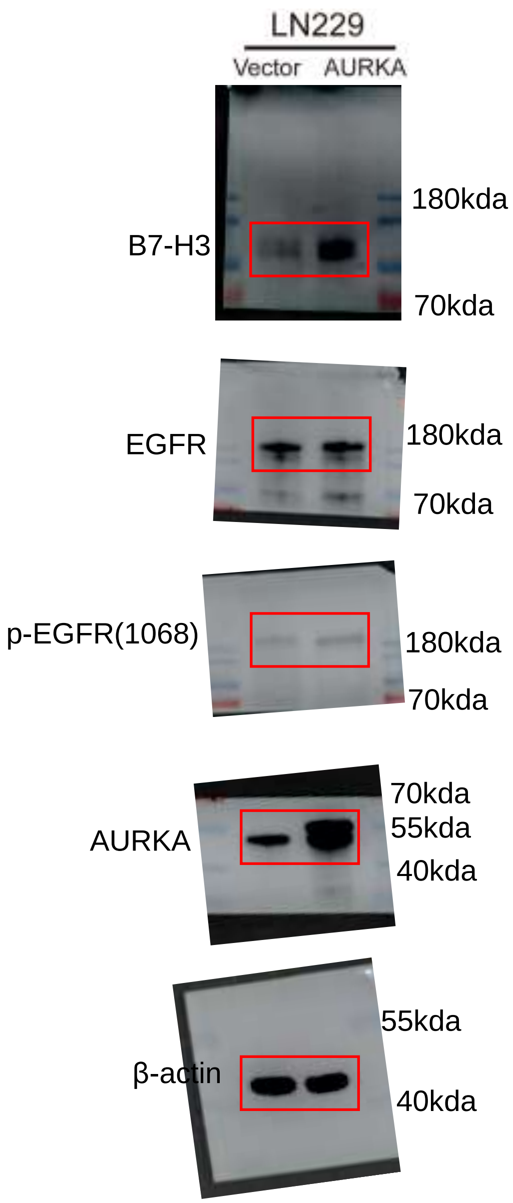

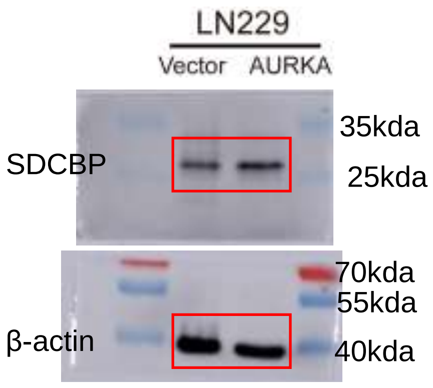

Figure 3A

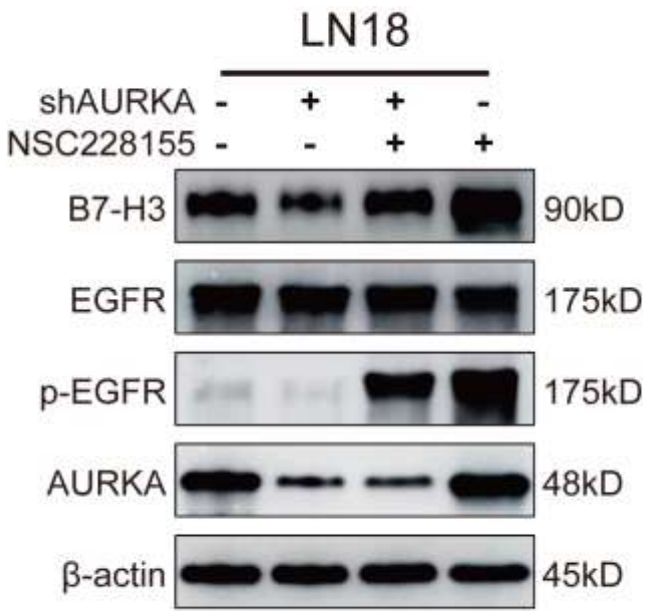

unedited gel

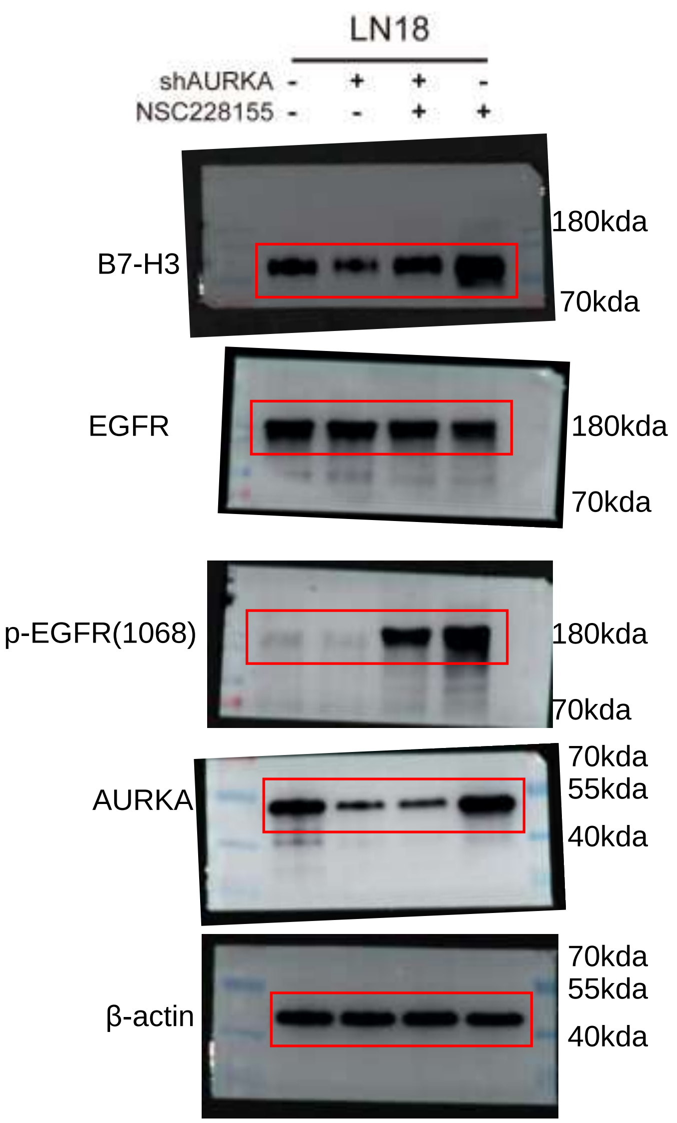

Figure 3D

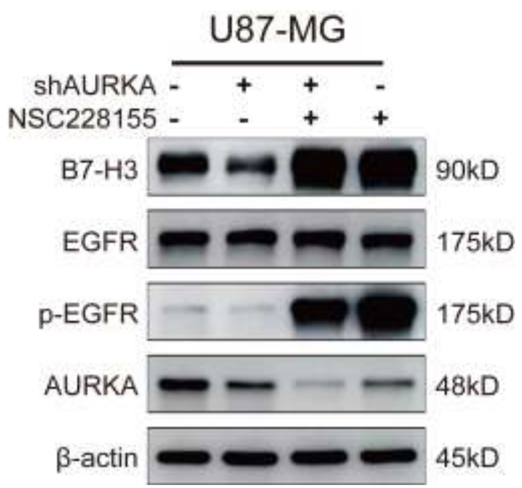

unedited gel

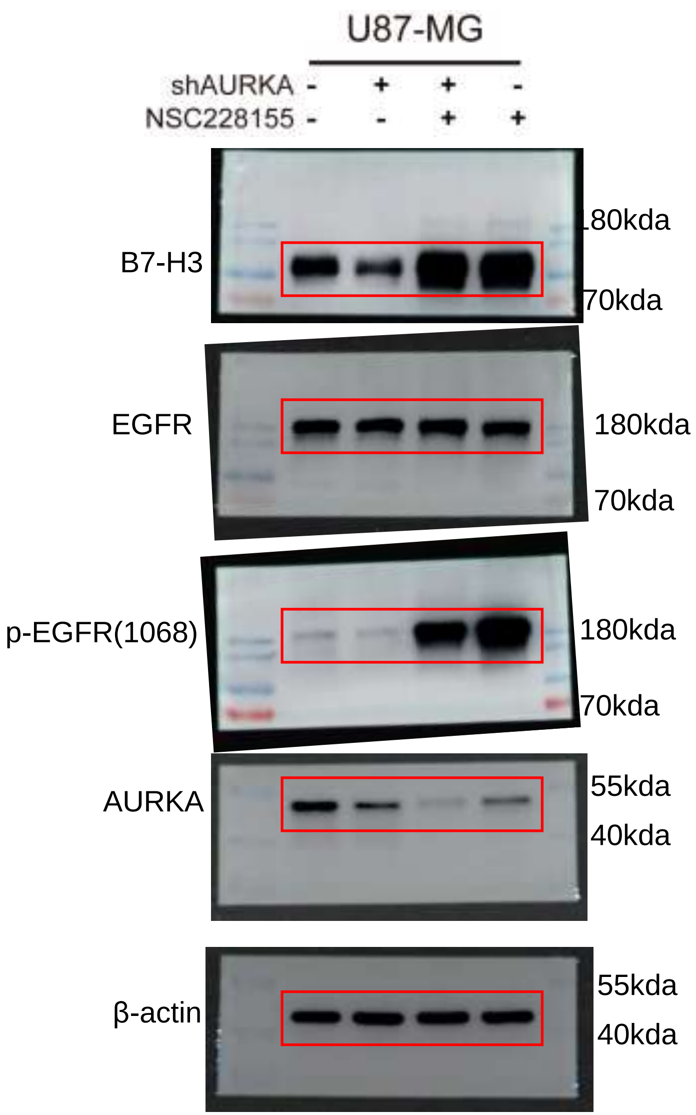

Figure 3G

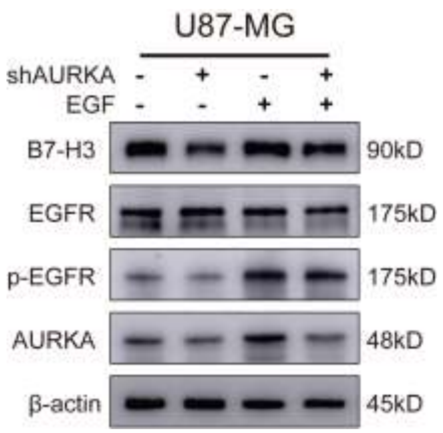

unedited gel

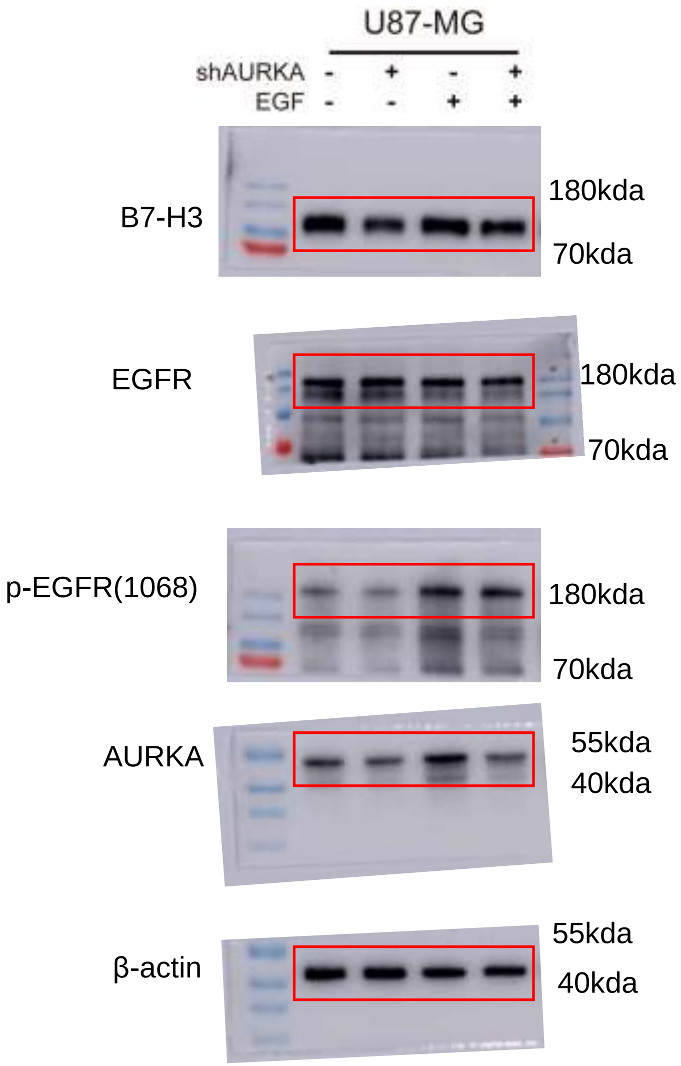

Figure 3I

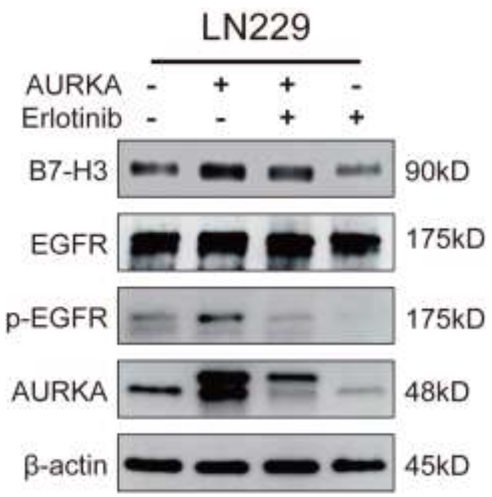

unedited gel

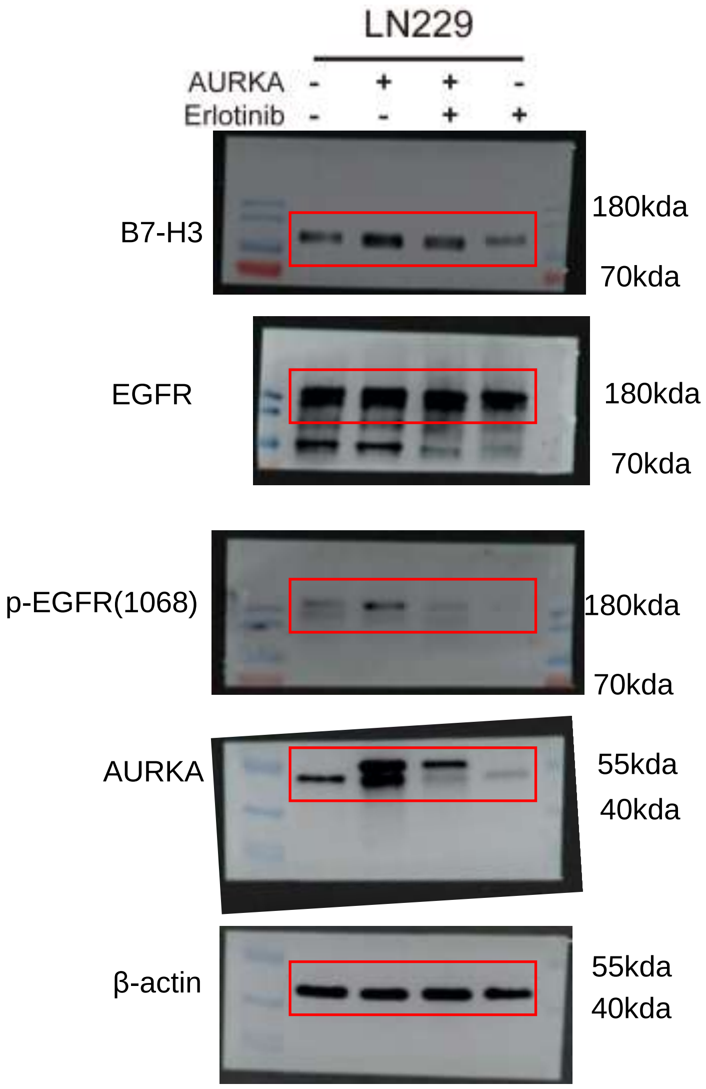

Figure 4A

unedited gel

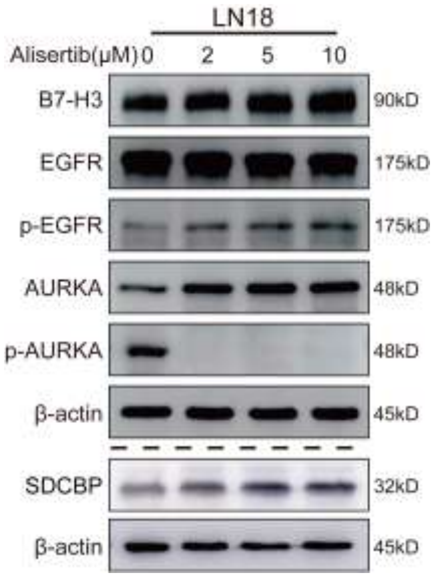

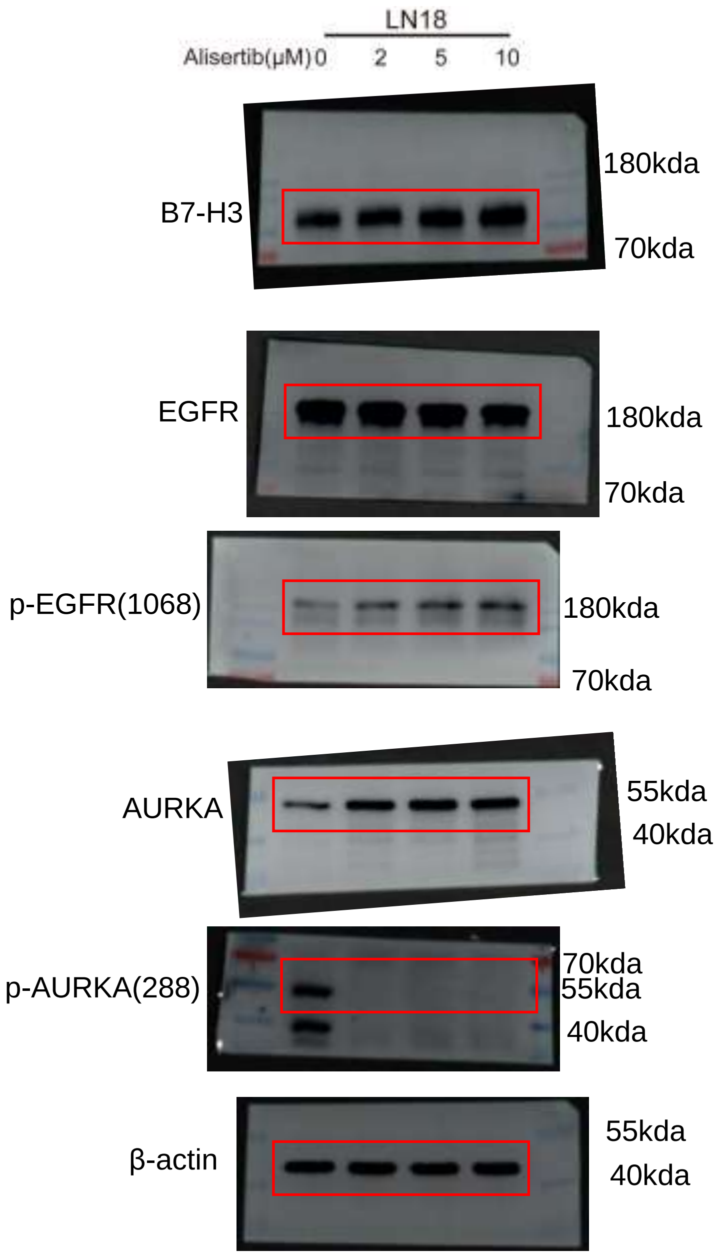

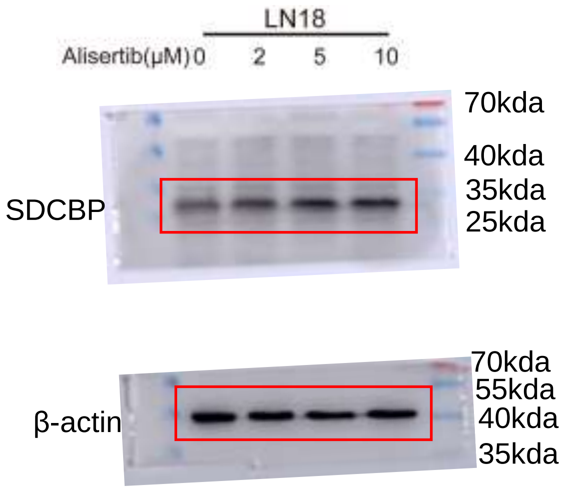

Figure 4B

unedited gel

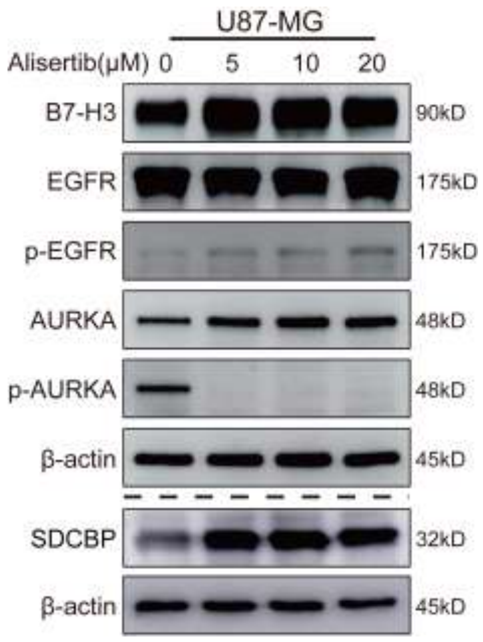

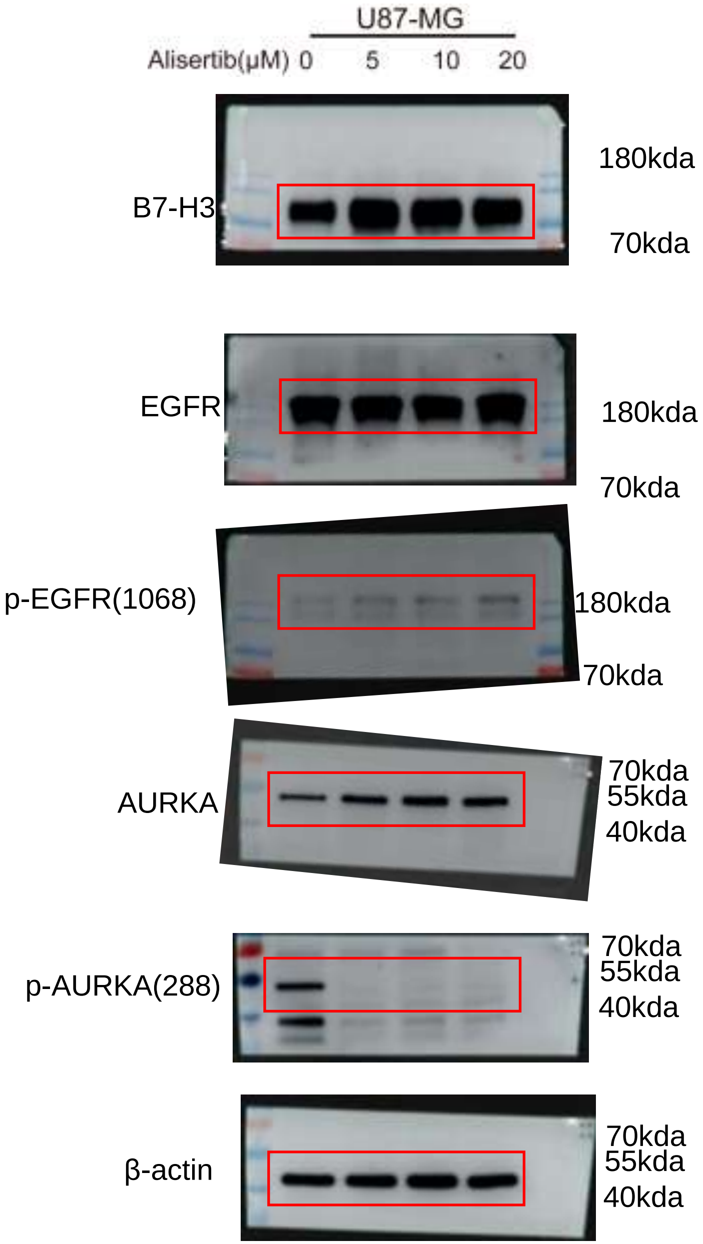

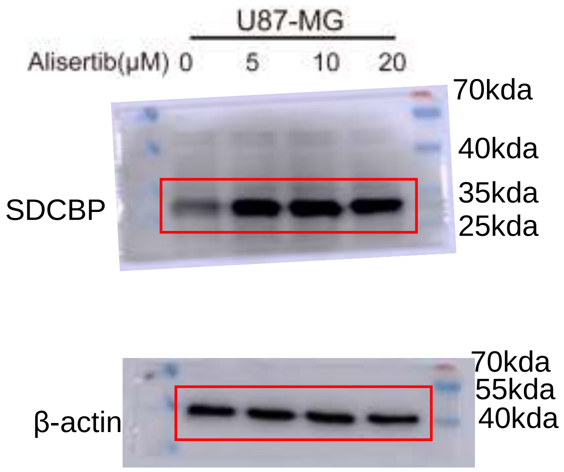

Figure 4C

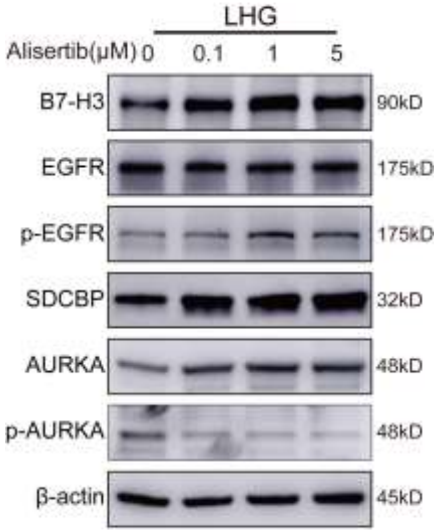

unedited gel

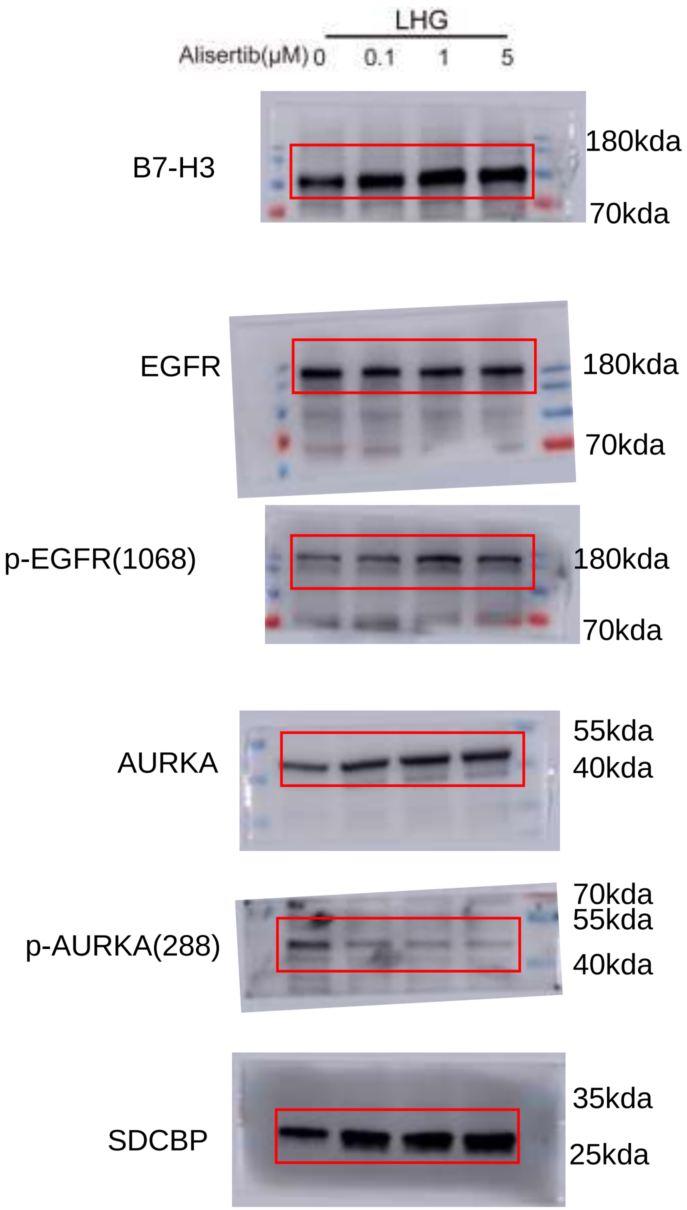

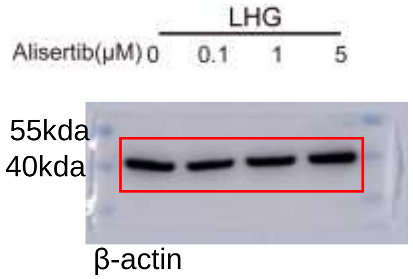

Figure 4D

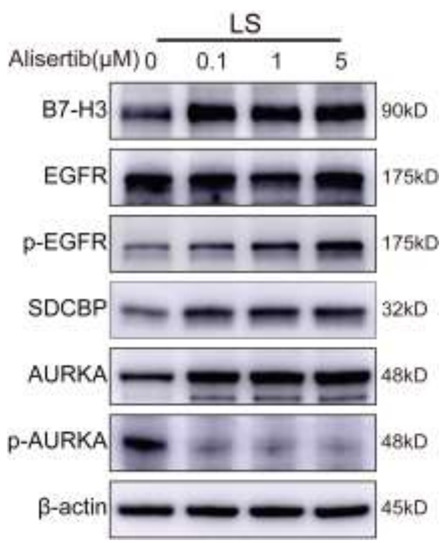

unedited gel

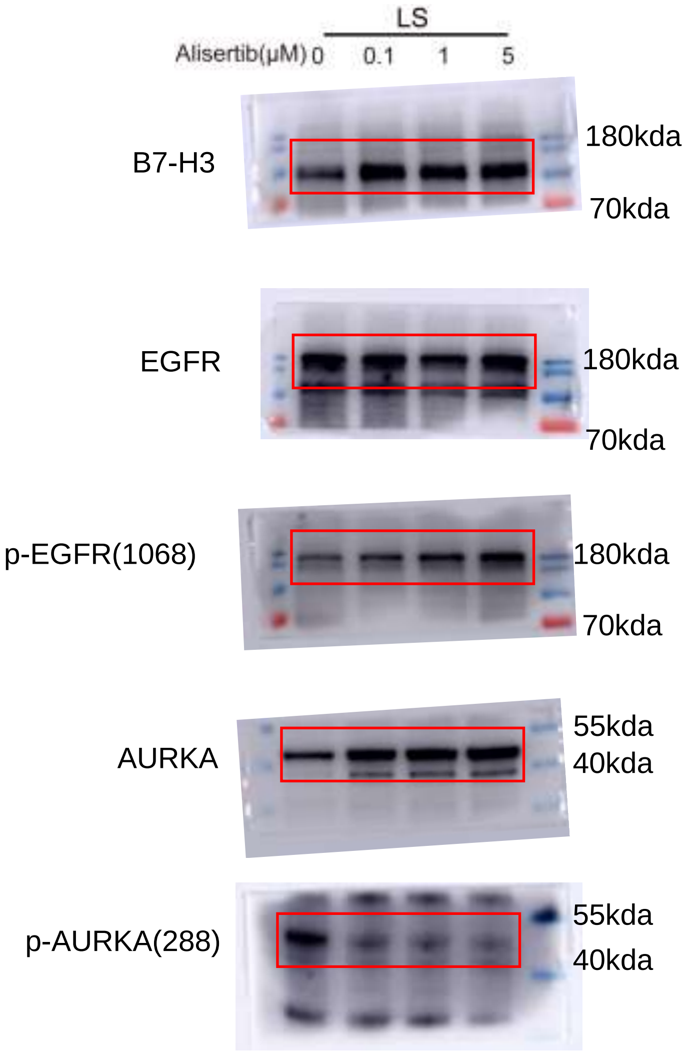

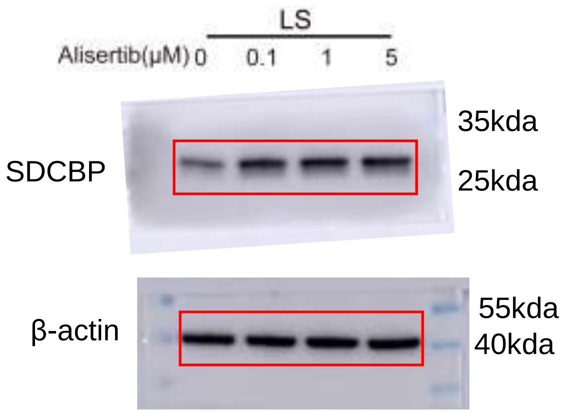

Figure 5A

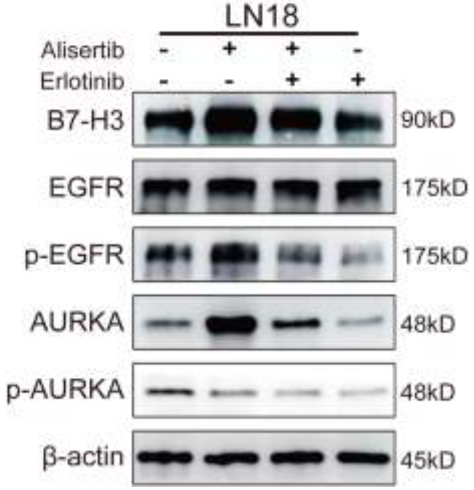

unedited gel

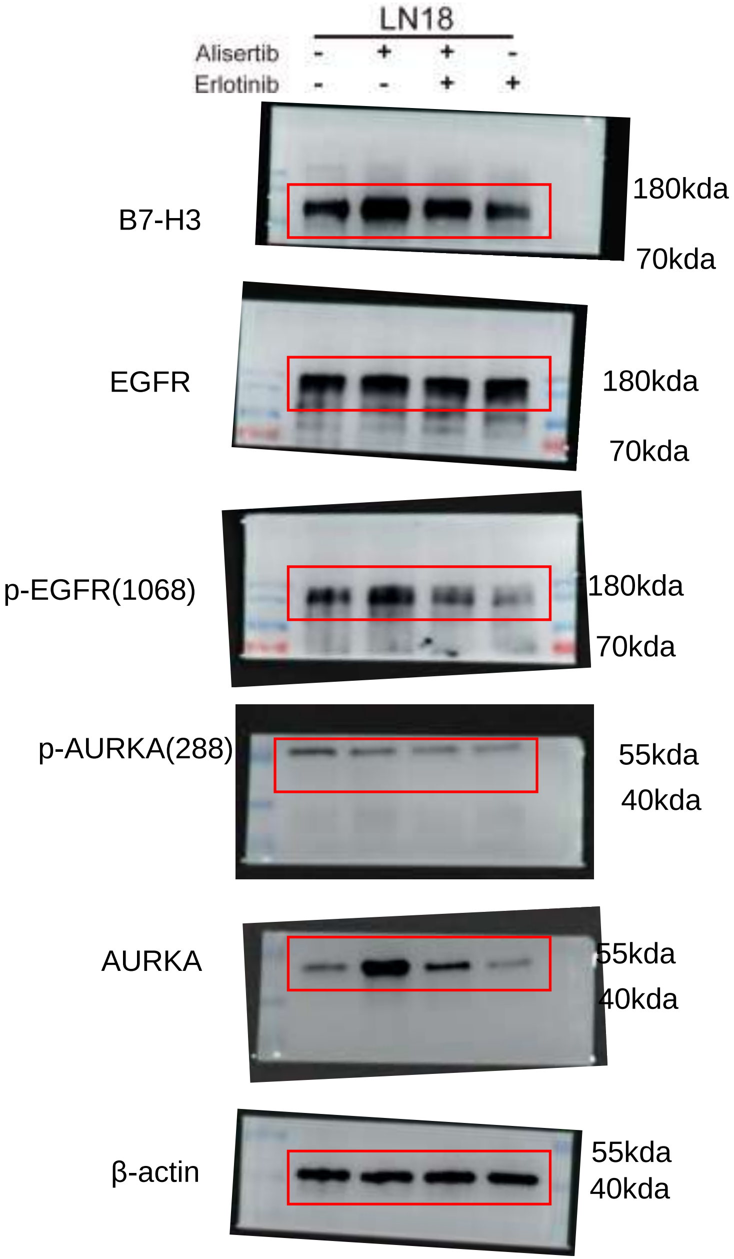

Figure 5D

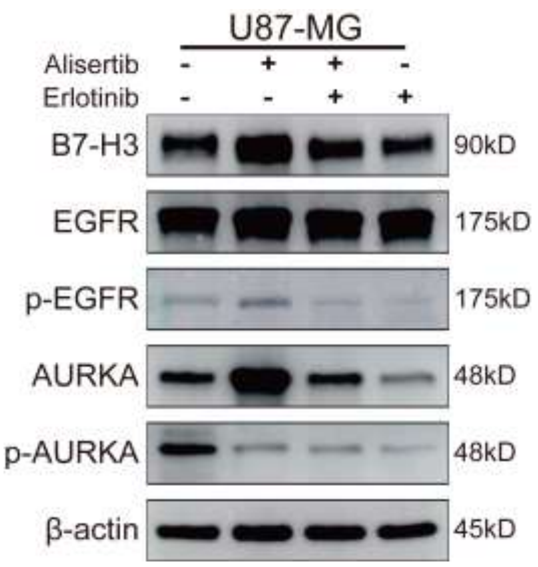

unedited gel

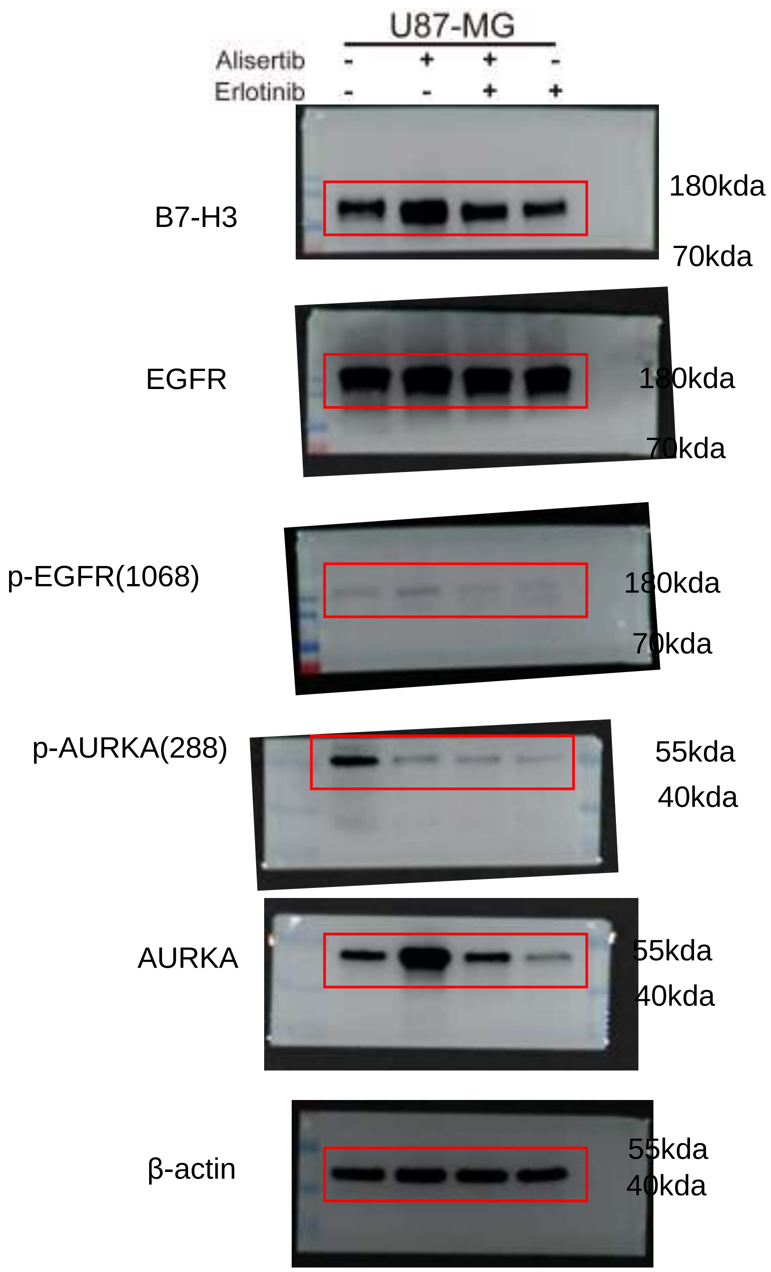

Figure S2A

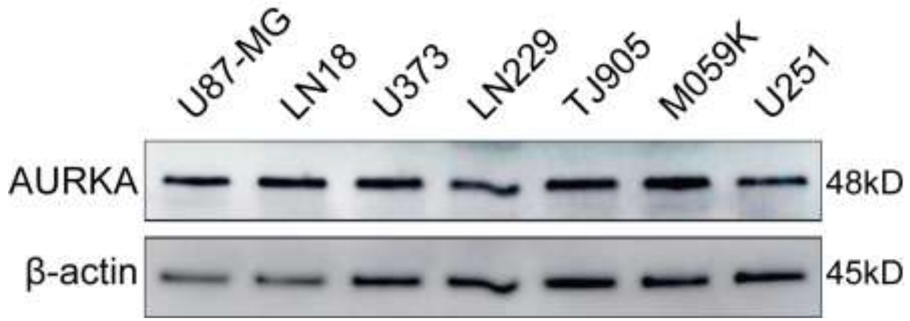

unedited gel

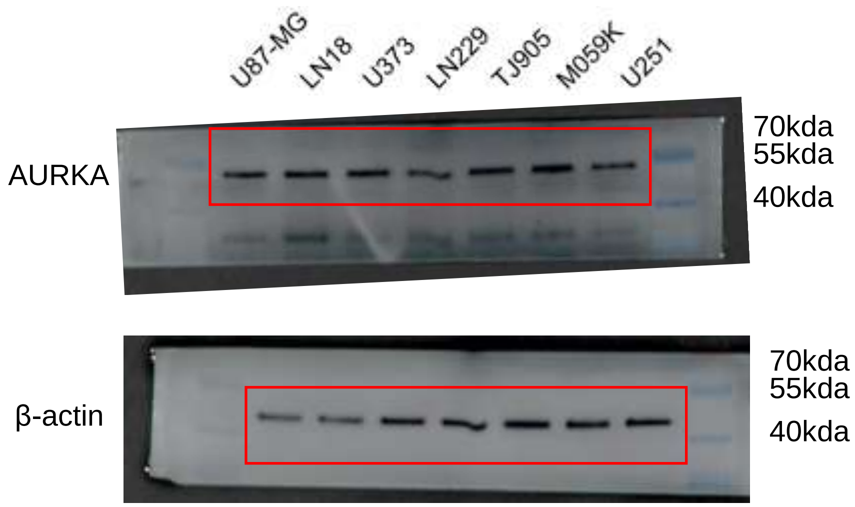

Figure S2B

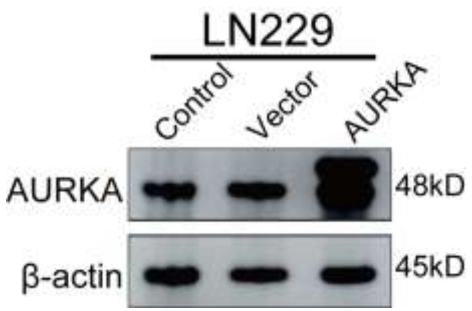

unedited gel

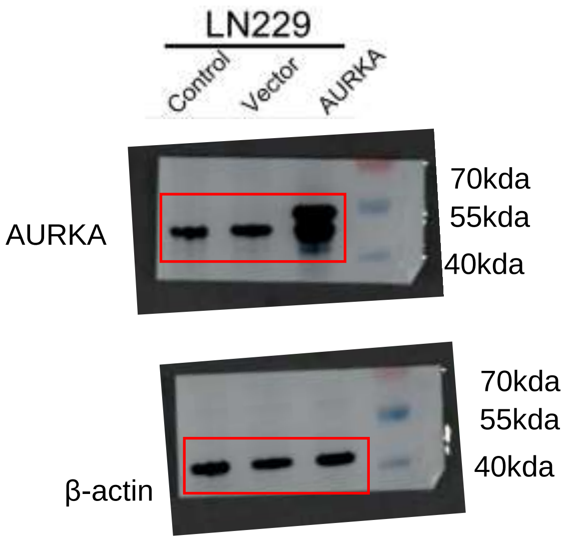

Figure S2C

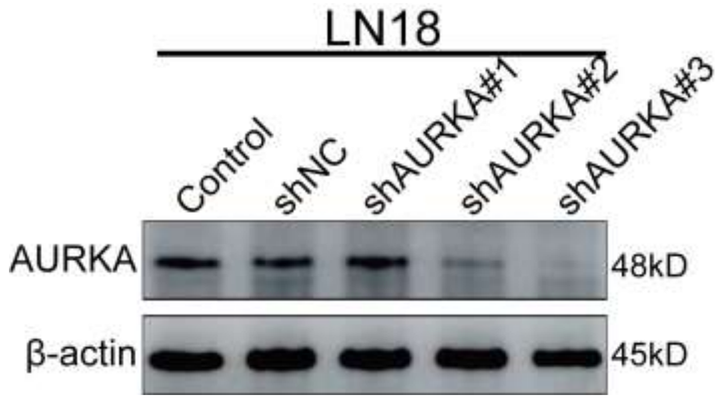

unedited gel

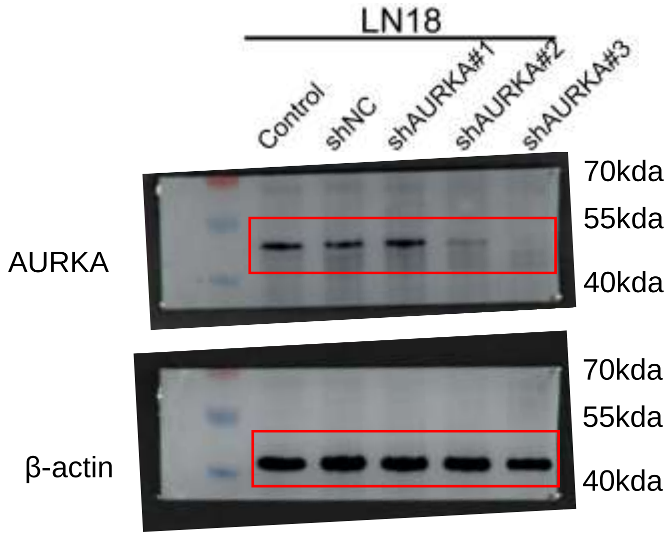

Figure S2D

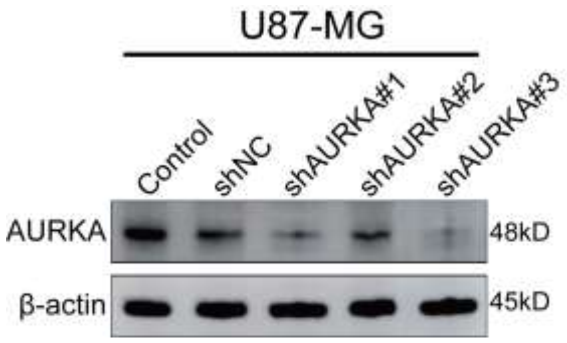

unedited gel

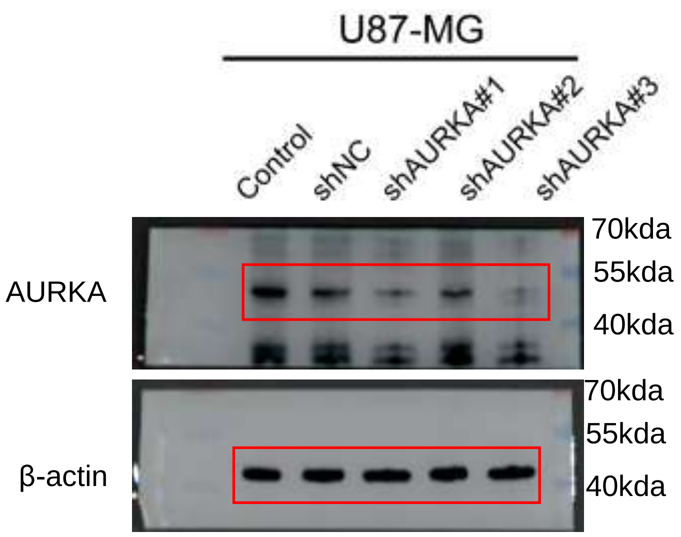

Figure S2F

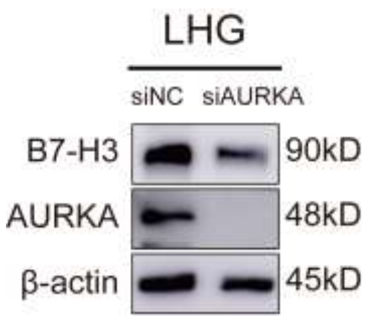

unedited gel

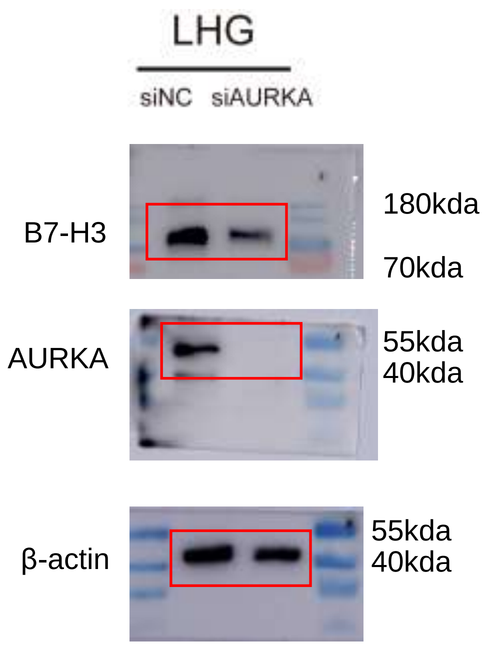

Figure S3C

unedited gel

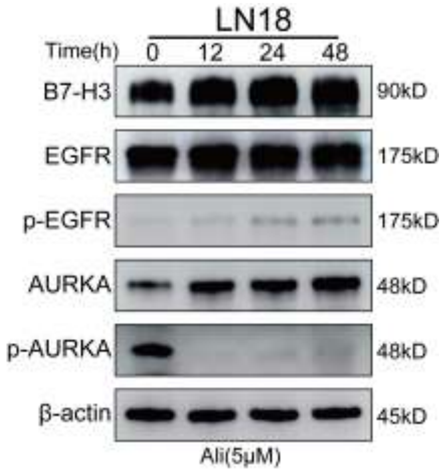

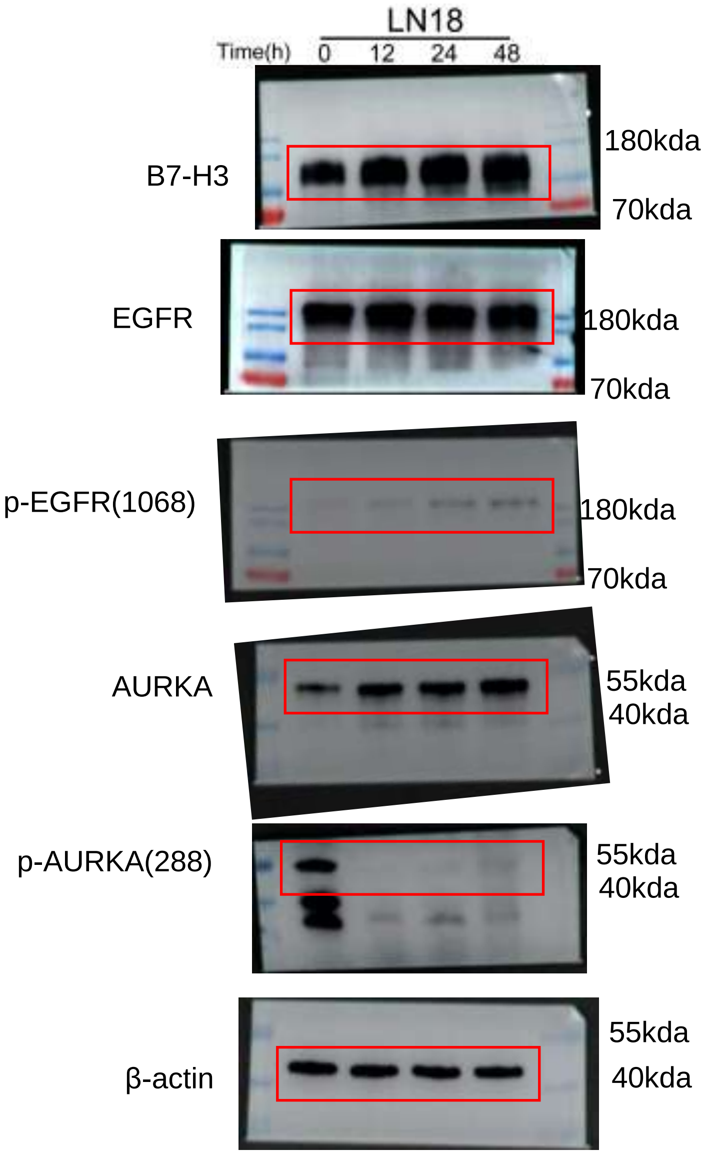

Figure S3D

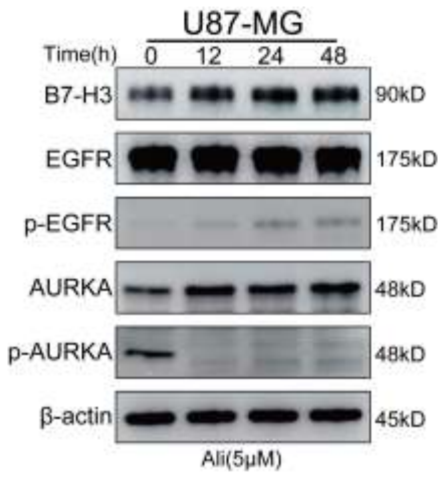

unedited gel

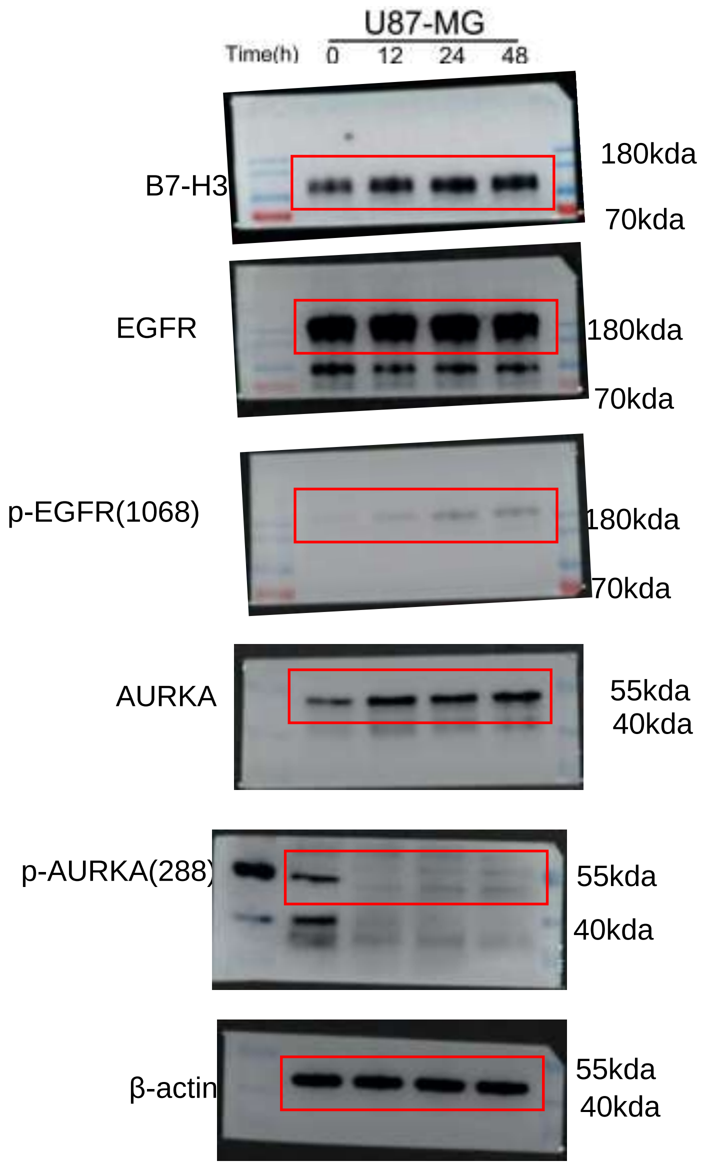

Figure S3G

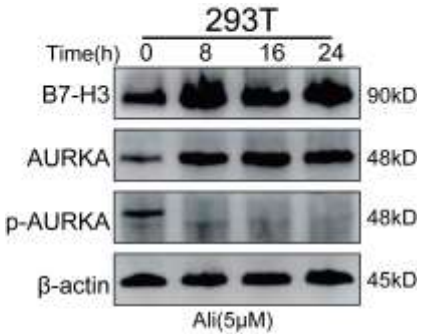

unedited gel

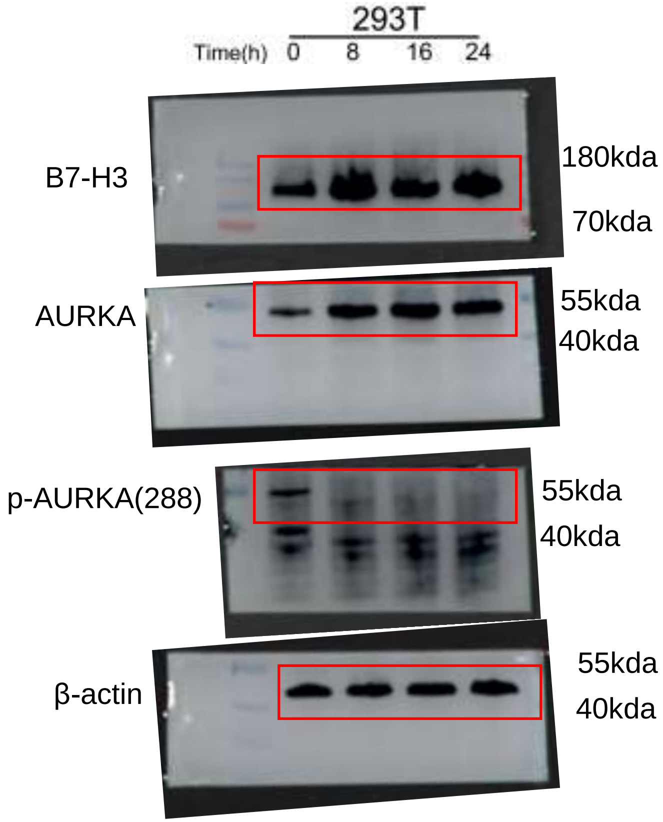

Figure S3I

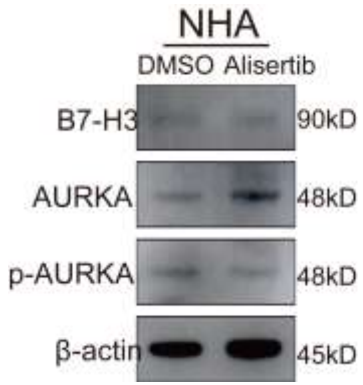

unedited gel

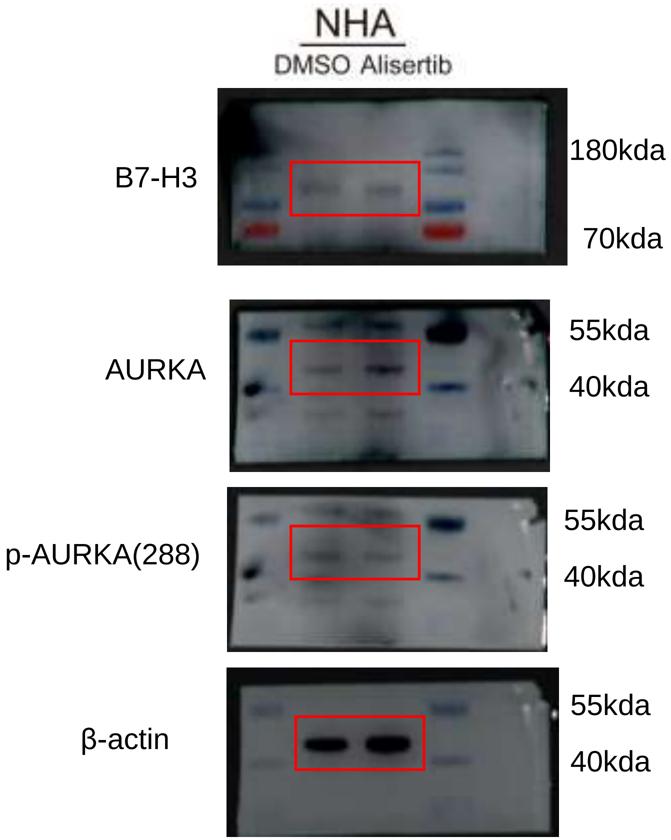

Figure S4B

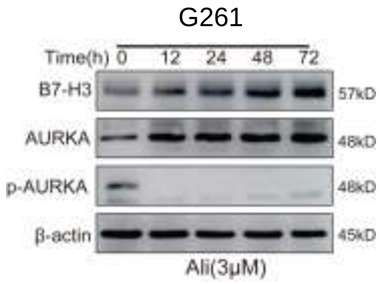

unedited gel

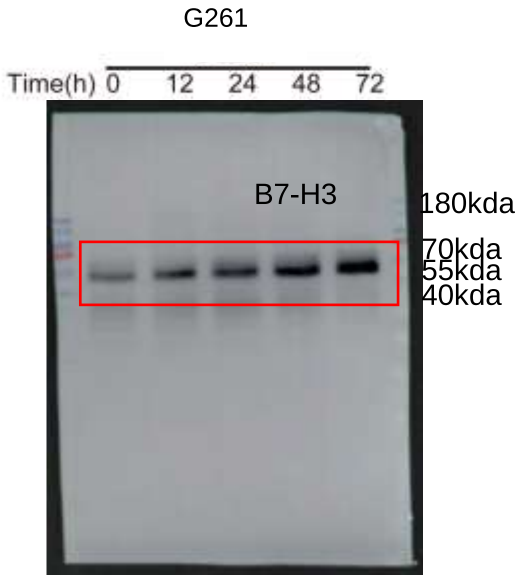

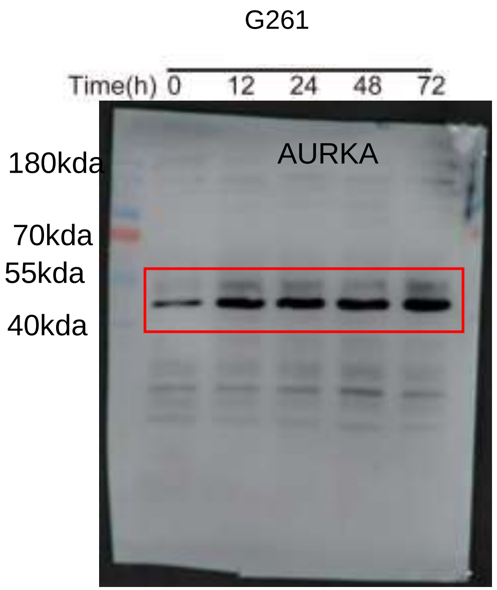

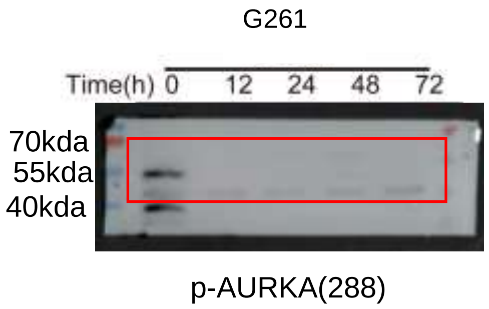

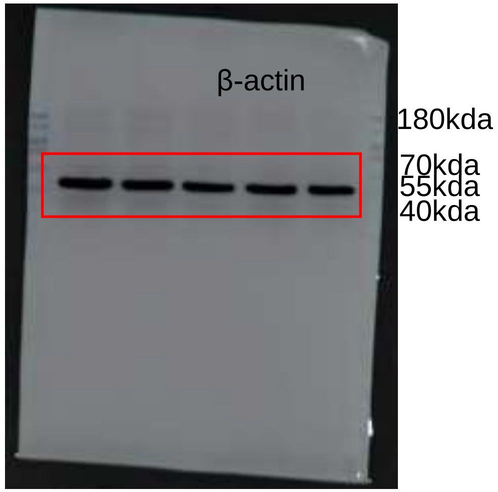

Figure S4C

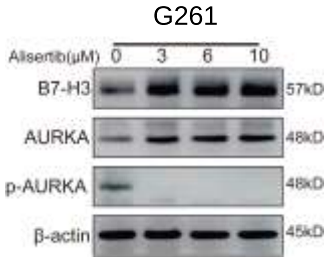

unedited gel

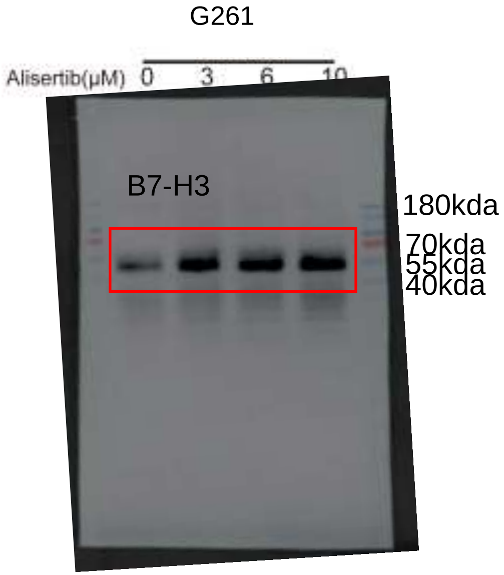

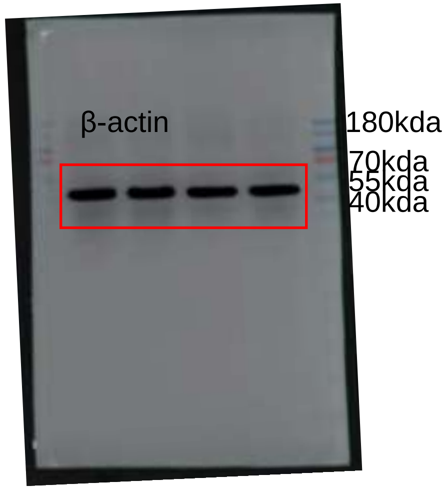

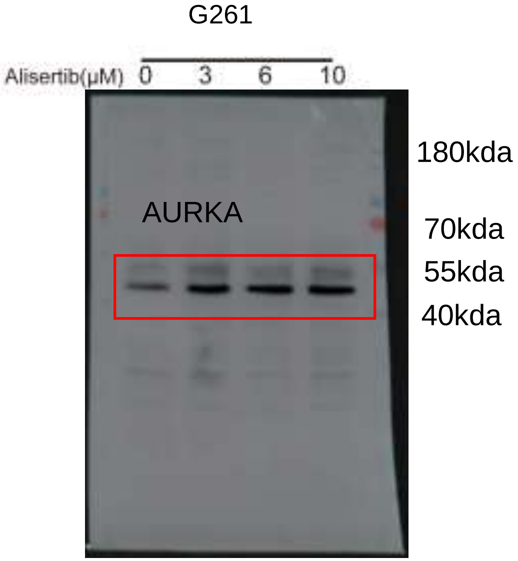

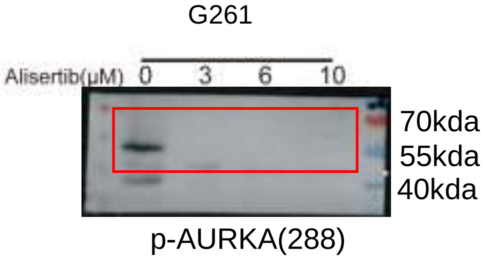

Supplement: Unedited blot and gel images [file jciinsight-10-173700-s123.pdf]
